# Machine Learning Profiling of Alzheimer's Disease Patients Based on Current Cerebrospinal Fluid Markers and Iron Content in Biofluids

**DOI:** 10.3389/fnagi.2021.607858

**Published:** 2021-02-22

**Authors:** Eleonora Ficiarà, Silvia Boschi, Shoeb Ansari, Federico D'Agata, Ornella Abollino, Paola Caroppo, Giuseppe Di Fede, Antonio Indaco, Innocenzo Rainero, Caterina Guiot

**Affiliations:** ^1^Department of Neurosciences “Rita Levi Montalcini”, University of Torino, Torino, Italy; ^2^Department NEUROFARBA, University of Firenze, Firenze, Italy; ^3^Department of Drug Science and Technology, University of Torino, Torino, Italy; ^4^Unit of Neurology 5 and Neuropathology, Fondazione Istituto di Ricovero e Cura a Carattere Scientifico Istituto Neurologico Carlo Besta, Milan, Italy

**Keywords:** cerebrospinal fluid, iron, biomarker (BM), mild cognitive impairment, Alzheimer's disease

## Abstract

Alzheimer's disease (AD) is the most common form of dementia, characterized by a complex etiology that makes therapeutic strategies still not effective. A true understanding of key pathological mechanisms and new biomarkers are needed, to identify alternative disease-modifying therapies counteracting the disease progression. Iron is an essential element for brain metabolism and its imbalance is implicated in neurodegeneration, due to its potential neurotoxic effect. However, the role of iron in different stages of dementia is not clearly established. This study aimed to investigate the potential impact of iron both in cerebrospinal fluid (CSF) and in serum to improve early diagnosis and the related therapeutic possibility. In addition to standard clinical method to detect iron in serum, a precise quantification of total iron in CSF was performed using graphite-furnace atomic absorption spectrometry in patients affected by AD, mild cognitive impairment, frontotemporal dementia, and non-demented neurological controls. The application of machine learning techniques, such as clustering analysis and multiclassification algorithms, showed a new potential stratification of patients exploiting iron-related data. The results support the involvement of iron dysregulation and its potential interaction with biomarkers (Tau protein and Amyloid-beta) in the pathophysiology and progression of dementia.

## Introduction

Alzheimer's disease (AD) is the most common cause of dementia (International, 2019), characterized by a complex etiology and unsatisfactory therapeutic approaches (Long and Holtzman, 2019). The duration of the preclinical and prodromal phase of AD varies from 10 to 20 years before clinical symptoms emerge (Vermunt et al., 2019). Mild Cognitive Impairment (MCI), which identifies a clinical condition that includes impairment in memory and/or non-memory cognitive domains, is assumed as a prodromal stage of AD, also referred to as MCI due to AD (Albert et al., 2011).

The presence of extracellular amyloid-beta (Aβ) deposition as neuritic plaques and of intracellular accumulation of hyperphosphorylated tau (p-Tau) as neurofibrillary tangles are the two hallmark lesions that histopathologically characterize the brains of AD patients (Ittner and Götz, 2011). However, there is evidence that significant accumulation of these pathological features can occur in non-demented individuals (Fagan et al., 2007; Aizenstein et al., 2008; Villemagne et al., 2008; Price et al., 2009) and also a high neuropathological heterogeneity is observed in patients with clinical diagnosis of AD (Rabinovici et al., 2016; Di Fede et al., 2018; Robinson et al., 2018). The abnormal concentration in the Cerebrospinal Fluid (CSF) of the proteins responsible for the plaque formation, i.e., Aβ42, p-Tau, and total-Tau (t-Tau), is assumed to be a measurable fingerprint of their brain deposition, reflecting neurochemical changes arising from AD pathology (Henry et al., 2013).

Although diagnostic criteria for AD and MCI are currently used (Dubois et al., 2007, 2010; Albert et al., 2011; McKhann et al., 2011; Sperling et al., 2011), defining the preclinical state of MCI/AD aiming at discovering therapies preventing the irreversible progression of AD (Fiandaca et al., 2014) remains a challenge.

New biomarkers and a deeper comprehension of the neuropathological processes involved in AD are urgently needed, with the aim to identify alternative disease-modifying therapies counteracting the disease progression. Actually, additional fluid biomarkers measured in CSF or in blood (Palmqvist et al., 2020) unraveled promising candidates, reflecting several inter-related mechanisms of AD pathophysiology (Molinuevo et al., 2018).

In this regard, a growing amount of evidence suggests the involvement of brain iron metabolism in the onset of several neurodegenerative diseases, in particular its accumulation in brain regions (Hare et al., 2013; Ward et al., 2014) and its potential key role in the pathogenesis of AD (Silvestri and Camaschella, 2008; Kozlov et al., 2017). Iron is an essential element for our body but, in spite of its ubiquity, it requires very careful managing (Gozzelino and Arosio, 2016). Free iron is a potentially toxic element that may generate Reactive Oxygen Species (ROS) triggering oxidative stress, lipid peroxidation, and DNA damage, also promoting cell death in the novel form of “ferroptosis”(Dixon et al., 2012; Hao et al., 2018). Therefore, most of the circulating and stored iron is linked to proteins and other transporters, such as transferrin (s-Tf) and ferritin (Pantopoulos et al., 2012; Eid et al., 2017). The systemic organs and the brain share the same iron regulatory mechanisms and pathways based on iron-modulating proteins, providing a link to the maintenance of iron homeostasis within the brain (Singh et al., 2014).

The imbalance in iron homeostasis in AD and its interaction with the more consolidated biomarkers Aβ and Tau have been described (Ndayisaba et al., 2019; Spotorno et al., 2020), supporting the conjecture of new therapeutic strategies based on iron chelators or other iron-toxicity counteracting drugs as a valuable approach for AD treatment (Liu et al., 2018; Ashraf and So, 2020). Several studies supported the notion that brain iron elevation (Lane et al., 2018; Ayton et al., 2019) or even the levels of iron-related proteins, e.g., plasma transferrin, are associated with AD and cognitive decline (Hare et al., 2015; Guan et al., 2020). Furthermore, the concentration of several elements included iron in biological fluids (Duce and Bush, 2010; Schrag et al., 2011; Cicero et al., 2017) has been evaluated with different techniques but highlighting difficulties to compare results. However, a direct evaluation of iron concentration in the brain remains a difficult task and conclusive results about the combined role of iron and iron-protein levels on biological fluids (i.e., CSF and serum) with the preclinical stage of dementia are not still clearly established.

Recently, the application of machine learning techniques gave strong support to AD diagnosis, in particular for classification tasks (Tanveer et al., 2020) and clustering analysis (Alashwal et al., 2019), aiming at identifying which features are involved in the conversion from early-stage AD to dementia. In particular, clustering analysis is a potentially strategic tool able to establish subsets of individuals sharing similar patterns and has been applied to investigate disease-related profiles of different AD and dementia stages (Racine et al., 2016; Alashwal et al., 2019).

This study aimed to investigate potential patterns of iron imbalances both in CSF and in serum, to improve early diagnosis and the related therapeutic possibility. While the content of iron in serum was assessed using standard clinical methods to detect transferrin (s-Tf), an accurate quantification of total iron in CSF was obtained using atomic absorption spectrometry, not currently used in clinical practice, leading to a potential added value to the clinical information about the status of the patients.

Firstly, to discriminate iron profiles between different forms of dementia, iron concentration in CSF of AD patients was compared with patients affected by Frontotemporal Dementia (FTD), a heterogeneous disorder with distinct pathological features and clinical phenotypes, encompassing changes in behavior, language, executive control, and often motor symptoms (Olney et al., 2017). Secondly, we compared patients affected by AD, MCI, and non-demented controls, to evaluate shared patterns and the ability to discriminate these conditions.

To check whether the new iron-related biomarkers could add significant improvements to AD early diagnosis, a step-by-step procedure was adopted, iteratively adding to the well-consolidated features (Aβ42, p-Tau, and t-Tau) also the results from s-Tf and iron content in CSF.

Cluster analysis was performed, to unravel subgroups within heterogeneous data such that individual clusters classify similar profiles, having better homogeneity than the whole. In particular, the hierarchical agglomerative clustering (HAC) algorithm was applied, a suitable technique for partitioning patients based on their similarity.

Since clustering analysis can reveal similar (pathological) profiles and identify potential altered biological mechanisms, we investigated how such clusters are influenced by the addition of the iron-related parameters and whether MCI can be better discriminated from controls and AD. Multiclassification algorithms with different features sets are used to compare diagnostic power and to rank the relevance of features for the prediction of the model.

## Materials and Methods

### Participants

We retrospectively included 69 patients (35 males and 34 females, mean age: 70.5 years ± SD: 7.2), evaluated and followed at the Department of Neurosciences of University Hospital “Città della Salute e della Scienza,” Torino and at Fondazione IRCCS Istituto Neurologico Carlo Besta, Milano, Italy.

CSF samples from all 69 patients, including 14 non-demented neurological control (CT) patients, 17 patients affected by MCI, 16 AD, and 22 FTD (behavioral variant) were collected.

Diagnosis of FTD was made according to Rascovsky Criteria (Rascovsky et al., 2011).

Diagnosis of AD has been made according to NIA-AA (National Institute of Aging—Alzheimer Association) criteria (McKhann et al., 2011).

For the classification of MCI, the Petersen criteria were used: cognitive complaint, decline or impairment; objective evidence of impairment in cognitive domains; essentially normal functional activities; not demented (Petersen, 2004; Petersen et al., 2009).

MCI group included a mix of amnestic, non-amnestic, and multidomain subjects, with disease onset before (*N* = 3) and after (*N* = 13) 65 years.

As control group, CSF of 14 patients with neurological conditions (see Supplementary Material) without dementia was analyzed.

Cognitive functions were assessed by Mini Mental State Examination (MMSE).

A complete description of data is available in Table 1. The experiments conformed to the principles of the Declaration of Helsinki and were approved by the local ethics committee. Informed consent for liquor collection and storage relative to the retrospective study was given by all subjects or by their caregivers.

**Table 1:** Numbers indicate frequency for gender and mean ± standard deviation for age, CSF biomarkers, MMSE, s-Tf.

	**CT** ***n =* 14**	**MCI** ***n =* 17**	**AD** ***n =* 16**	**FTD** ***n =* 22**	**Statistical** **analysis**
Gender (M/F) (Count)	10/4	8/9	7/9	10/12	NS
Age at the time of CSF collection (yrs) (mean ± SD)	(72.13 ± 6.96)	(72.36 ± 4.11)	(68.47 ± 7.38)	(69.45 ± 8.69)	NS
	**CT** ***n =*** **14**	**MCI** ***n =*** **17**	**AD** ***n =*** **16**	**FTD** ***n =*** **22**	
Age onset (mean ± SD)	(68.64 ± 7.16)	(66.82 ± 6.57)	(65.87 ± 7.20)	(65.91 ± 8.11)	NS
Aβ42 CSF (pg/mL) (mean ± SD)	(917.93 ± 277.15)	(832.65 ± 399.16)	(432.00 ± 200.68)	(819.45 ±322.91)	AD < CT: (*p* < 0.001) ^†^, AD < MCI: (p=0.002) ^†^, AD < FTD: (*p* < 0.001) ^†^
p-Tau CSF (pg/mL) (mean ± SD)	(19.9 ± 8.91)	(57.47 ± 58.14)	(84.65 ± 45.18)	(41.12 ± 23.92)	AD > CT: (*p* < 0.001) ^†^, AD > FTD: (*p* = 0.04) ^†^
t-Tau CSF (pg/mL) (mean ± SD)	(102.76 ± 72.24)	(326.18 ± 363.71)	(465.81 ± 329.45)	(154.53 ± 102.28)	AD > CT (p=0.002) ^†^, AD > FTD: (p=0.03) ^†^
	**CT** ***n =*** **10**	**MCI** ***n =*** **12**	**AD** ***n =*** **16**	**FTD** ***n =*** **17**	
MMSE (mean ± SD)	(26.54 ± 3.19)	(24.47 ± 5.16)	(20.63 ± 6.08)	(22.07 ± 4.55)	AD < CT (*p* = 0.03) ^†^
	**CT** ***n =*** **9**	**MCI** ***n =*** **9**	**AD** ***n =*** **11**	**FTD** ***n =*** **11**	
s-Tf (mg/dL) (mean ± SD)	(231.44 ± 38.01)	(230.78 ± 34.22)	(235.55 ± 17.99)	(255.72 ± 57.11)	NS

*AD, Alzheimer's disease; Aβ42, forty-two amino acid-long amyloid-β peptide; CSF, cerebrospinal fluid; CT, neurological control; F, female; FTD, Frontotemporal dementia; M, male; MCI, mild cognitive impairment; MMSE, Mini-Mental State Examination; NS, Not significant; p-Tau, hyperphosphorylated tau; s-Tf, serum transferrin; t-Tau, total tau*.

† *Kruskal-Wallis test and Dunn's post-hoc test (Bonferroni correction for p-value)*.

Details of procedure for the collection of CSF and serum samples, also with determination of CSF levels of Aβ42, p-Tau, and t-Tau and of serum transferrin, are reported in Supplementary Material.

### Iron Determination in CSF by GF-AAS

Frozen aliquots of CSF samples were transported on dry ice until the shipment to the analytical chemistry laboratory, were kept frozen during storage and unfrozen 1 h before the analysis.

The determination of iron in CSF samples was carried out by means of Graphite Furnace Atomic Absorption Spectrometer (GF-AAS) as detailed in Supplementary Material.

### Statistical and Machine Learning Analysis

The assumption of equality of variance and normal distribution were assessed through Levene's test and Shapiro-Wilk's test, respectively. One-way analysis of variance (ANOVA) for normally distributed variables, Kruskal-Wallis test for variables not following a normal distribution, and chi-squared test (for categorical variables) were conducted to determine group differences. *Post-hoc* tests (*t*-test and Dunn's test adjusted for multiple comparisons errors according to Bonferroni) were performed, respectively, after significant results of ANOVA and Kruskal-Wallis test. The same procedures were applied to compare clusters in the cluster analysis, described below.

Bivariate correlations between clinical data, biomarkers, and the iron concentration levels were tested both using Spearman's test and Pearson's test for non-parametric and parametric relationships (*r*_s_ = Spearman's rank correlation coefficient, *r* = Pearson's correlation coefficient), respectively. We assumed as correlated only the variables simultaneously satisfying the two correlation criteria, with both |r| and |r_s_|>0.5. Results from statistical analysis were evaluated against a threshold of *p* < 0.05.

Before the cluster analysis, Hopkins's test was applied to assess the clustering tendency of the datasets. For the hierarchical clustering, the clustering variables were selected based on the results of bivariate correlations, avoiding to include features with a high degree of collinearity (a threshold of *r* > 0.7 was set Dormann et al., 2013). For the variables presenting some association with age, additional analysis was performed including the age correction in the clustering analysis (Supplementary Material).

HAC was applied, a bottom-up approach in which each data point starts in a separate cluster, and pairs of clusters are merged at the bottom going up the hierarchy. After data standardization (Z-score unit), patients were grouped using HAC with Ward's method of minimum variance and Euclidean distance metric and visualized in dendrograms. Ward's method joins two clusters to make the smallest increases in the pooled within-cluster variation (Ward, 1963).

The number of resulting clusters was set finding clustering step where the acceleration of distance growth is the largest, stopping the process and selecting a distance cut-off in the dendrogram to determine the correct number of clusters (>2 clusters). Different sets of features in two datasets were considered for clustering, and a heatmap was used to visualize the median value of the features in each cluster. The values of features within each cluster are reported for the different feature sets used (Supplementary Material).

The clusters obtained for each feature set were compared based on the following clustering scores: Adjusted Rand Index (ARI) and Adjusted Mutual Information (AMI), measuring the similarity and agreement of the two assignments; V-measure, evaluating the homogeneity, and completeness of the clusters. In the subpopulation in which all features considered are available, the ratio (Fe CSF/s-Tf) was calculated for each cluster and the dataset was divided into quartiles to observe where the values of variables in each cluster fall with respect to the whole population (Supplementary Material).

The dataset was used to train two machine learning models based on Support Vector Machine (SVM) and Logistic Regression (LR) adapted for multiclass classification, using two different sets of features, comparing the performance of the classifiers and ranking the relevance of features. The SVM algorithm is very popular for discrimination tasks because it is able to reach good generalization ability and accurately combines features, finding the maximal margin hyperplane, and minimizing the classification error to divide data belonging to different classes (Cortes and Vapnik, 1995).

Two feature sets used for the cluster analysis have been included in the model. After standardization of the datasets, an exhaustive search over parameter values for the estimators has been carried out by cross-validated grid search to optimally tune parameters of the classifiers.

For the present study, the OnevsRest (OVR) classifiers based on SVM with linear kernel and LR were used for the classification of the three groups (CT, AD, MCI) and to evaluate the importance assigned to the features. The classification performance of the constructed models, varying the input features presented to it, was computed using the macro-averaged Area Under the Receiving Operating Curve (AUROC). The performance of the classifiers was assessed via 100 times stratified shuffle split cross-validation method (proportion of train:test size = 60:40). This cross-validation method returns stratified randomized folds that preserve the percentage of samples for each class.

The values of the importance for each feature were obtained applying the model inspection technique based on repeated permutations of features on test datasets. The permutation feature importance is defined to be the change in a model score when a single feature value is randomly shuffled. This procedure indicates how much the model depends on the feature, breaking the relationship between the feature and the target, correcting possible bias of the model.

Statistical and machine learning analysis was carried out under the programming language Python, also using library Scikit-Learn (Pedregosa et al., 2011).

## Results

### Demographic and Clinical Data

Demographic and clinical data of the patients classified by clinical diagnosis as described in section Materials and Methods 2.1 are reported in Table 1 (see also Supplementary Figure 1). Values of MMSE and s-Tf are available for different subgroups of the population composed of 69 patients.

In addition, the four groups were not significantly different for values of glucose and protein dosed in CSF (data not shown) and there were not significant differences in iron variables (s-Tf and iron in CSF) between men and women.

The variables p-Tau and t-Tau showed a strong positive correlation both for the whole population (*r*_s_ = 0.67, *r* = 0.87, *p* < 0.001) and for the population including only AD, MCI, and CT patients (*r*_s_ = 0.76, *r* = 0.92, *p* < 0.001). P-Tau and t-Tau indicated positive correlations also considering AD (*r*_s_ = 0.81, *r* = 0.88, *p* < 0.001), MCI (*r*_s_ = 0.61, *p* = 0.01, *r* = 0.91, *p* < 0.001), and CT (*r*_s_ = 0.73, *p* = 0.003, *r* = 0.66, *p* = 0.002) groups. Considering the subpopulation (s-Tf available) the strong correlation between p-Tau and t-Tau was confirmed (*r*_s_ = 0.89, *r* = 0.92, *p* < 0.001). In AD group s-Tf resulted negatively correlated with t-Tau (*r*_s_ = −0.70, *p* = 0.03, *r* = −0.67, *p* = 0.02). Additional results are reported in Supplementary Material.

### Iron Concentration in CSF

**Figure 1:**
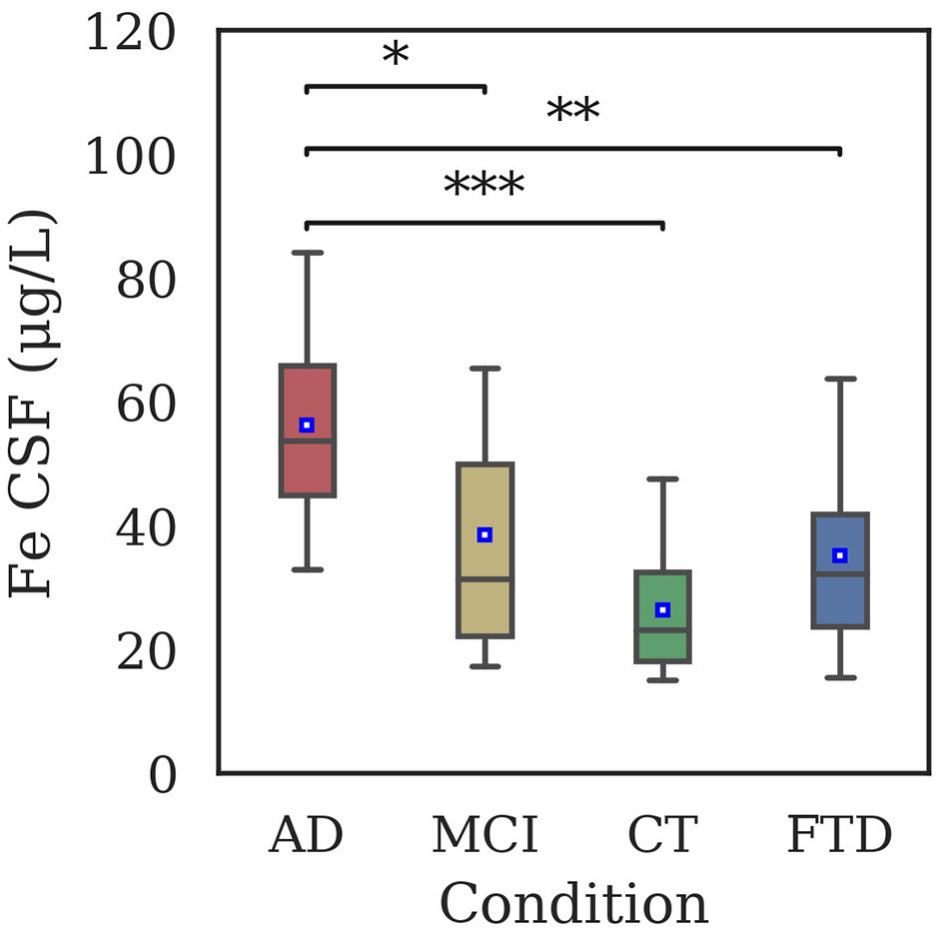
Distribution of iron concentration in CSF samples of AD (56.3 ± 15.6 μg/L), MCI (38.6 ± 21.2 μg/L), CT (26.5 ± 9.9 μg/L), and FTD (35.2 ± 16.8 μg/L) patients by means of GF-AAS. Iron levels were significantly different between AD and CT (*p* < 0.001), AD and FTD (*p* = 0.003), and AD and MCI (*p* = 0.02). Statistical differences have been evaluated by means of Kruskal-Wallis test and Dunn's *post-hoc* test using Bonferroni correction for *p*-value (^*^p < 0.05; ^**^p < 0.01; ^***^p < 0.001).

Total iron concentration in CSF samples of patients is shown in Figure 1. Significant differences have been reported between AD and all the other groups, but not between CT and MCI. No difference was found between FTD and CT groups.

### Clustering Analysis

Clustering analysis was performed including CT, MCI and AD groups on two different populations: total dataset comprising 47 patients, and the subset composed of 29 patients for which the measurement of s-Tf is available. The sets of features used to compare the results are the following:

- Standard Biomarkers dosed in CSF (SBs) (p-Tau, Aβ42);
- SBs + Iron in CSF
- SBs + s-Tf
- SBs + s-Tf + Iron in CSF

For the application of hierarchical clustering we dropped t-Tau, due to its high correlation with p-Tau.

#### SBs

**Figure 2:**
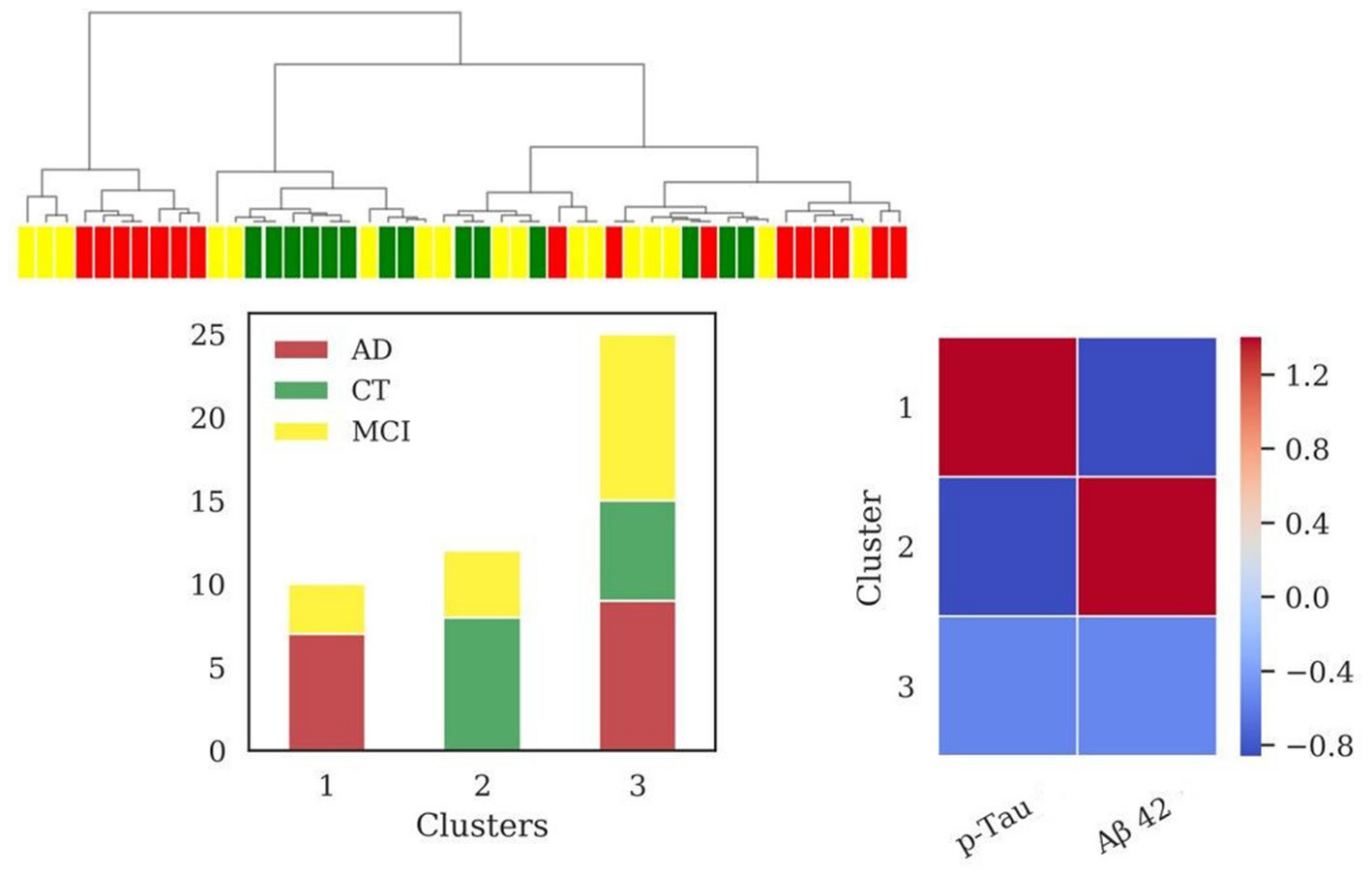
Results of hierarchical clustering using standard biomarkers Aβ42 and p-Tau, dosed in CSF. Left: Dendrogram (yellow = MCI; red = AD; green = CT) and distribution of patients within the three clusters. Right: Heatmap using the median value of the features (Z-score unit) in each cluster. AD, Alzheimer's Disease; CT, neurological control; MCI, Mild Cognitive Impairment.

HAC based on standard biomarkers (p-Tau, Aβ42) (Figure 2) showed the emergence of three clusters (sizes: *N* = 10, *N* = 12, *N* = 25). Differences in Aβ42 was very significant (*p* < 0.001) between clusters 1 and 2 and cluster 2 and 3, while differences in p-Tau concentration (*p* < 0.001) between clusters 1 and 2 and clusters 1 and 3. Values of MMSE have been calculated for each cluster [cluster 1 = (20.9 ± 6.3); cluster 2 = (25.1 ± 4.9); cluster 3 = (24.1 ± 5.4)]. External scores have been evaluated for the clustering: V-measure (0.22), ARI (0.09), AMI (0.18).

#### SBs + Iron in CSF

**Figure 3:**
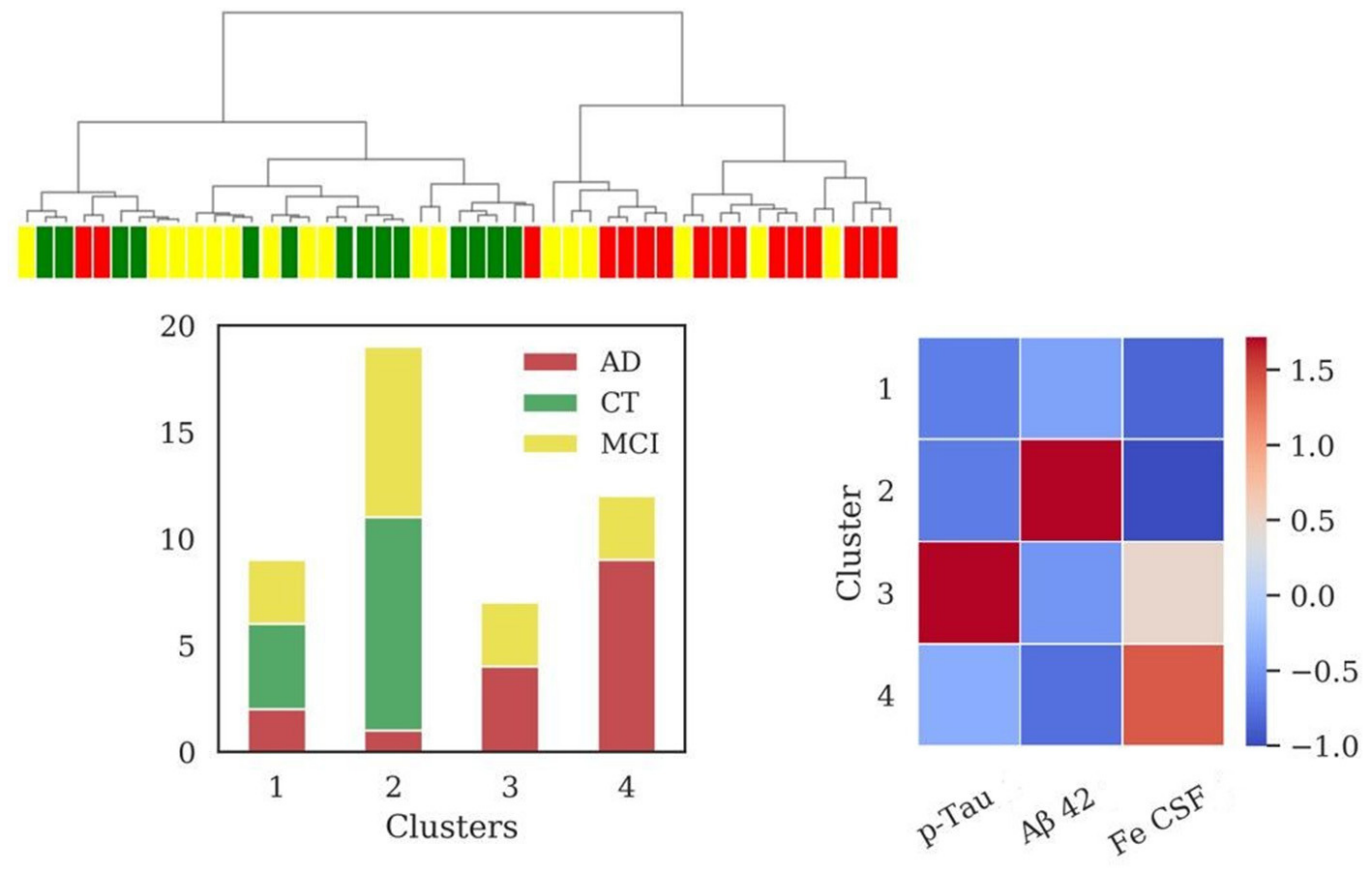
Results of hierarchical clustering using biomarkers and iron concentration in CSF. Left: Dendrogram (yellow = MCI; red = AD; green = CT) and distribution of patients within the four clusters. Right: Heatmap using the median value of the features (Z-score unit) in each cluster. AD, Alzheimer's Disease; CT, neurological control; MCI, Mild Cognitive Impairment.

The addition of iron dosage in CSF unraveled four clusters (sizes: *N* = 9, *N* = 19, *N* = 7, *N* = 12) after the application of HAC, reported in Figure 3. The clusters composed of AD and MCI patients (cluster 3 and cluster 4) significantly differed among them for iron (*p* = 0.038) and p-Tau (*p* < 0.001) profiles and from cluster 2 (mainly composed of CT patients) for all variables with high significance (*p* < 0.001), except for p-Tau between cluster 2 and 4 (*p* = 0.018). Cluster 3 differed from cluster 1 for CSF iron content (*p* = 0.006).

Values of MMSE have been calculated for each cluster [cluster 1 = (24.7 ± 4.8); cluster 2 = (25.2 ± 4.5); cluster 3 = (22.1 ± 4.7); cluster 4 = (21.6 ± 7.3)].

The addition of CSF iron improved V-measure (0.25), ARI (0.16), and AMI (0.20) with respect to the same scores obtained with the biomarkers set, described in the previous section.

#### SBs + s-Tf

**Figure 4:**
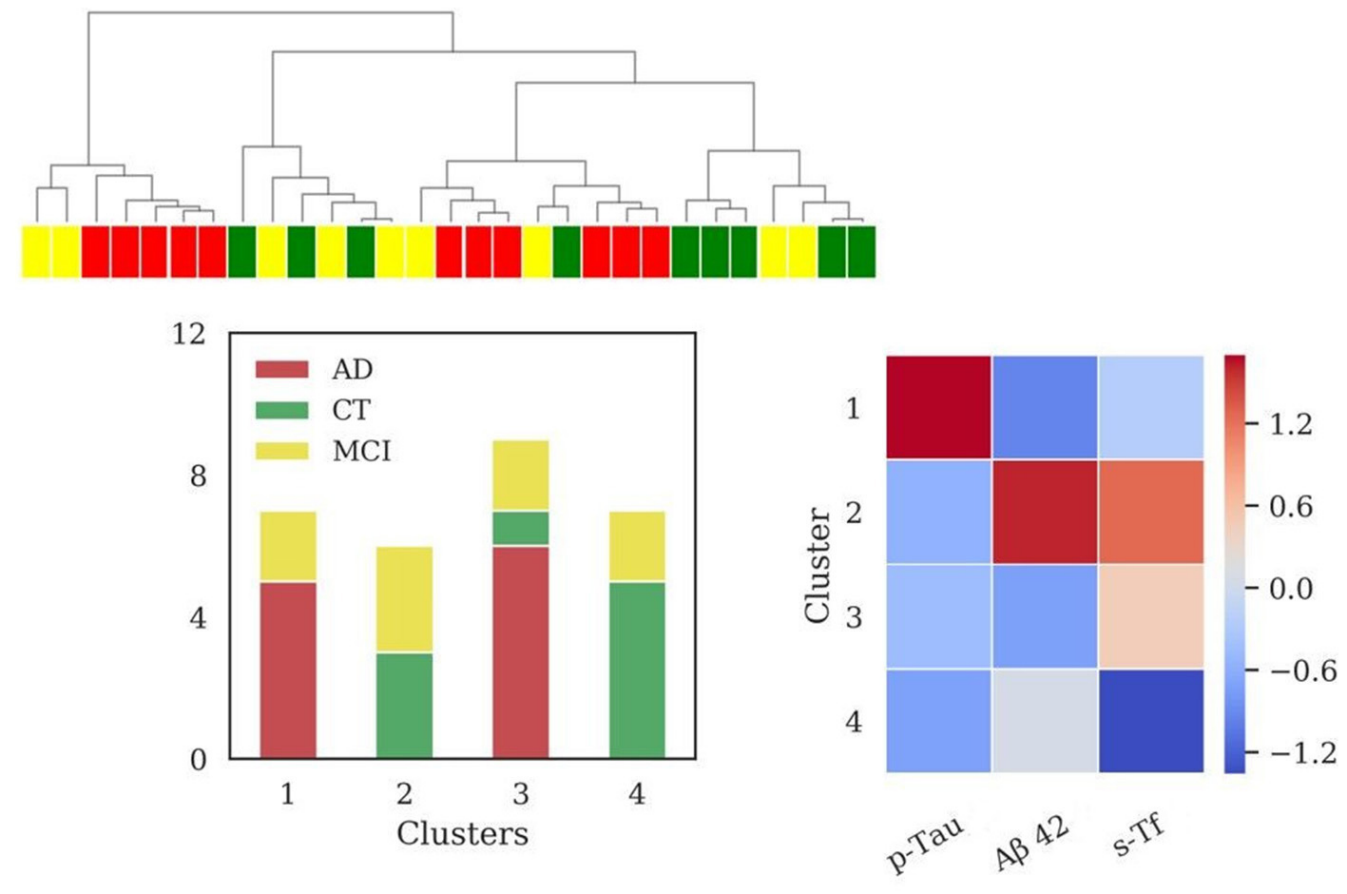
Results of hierarchical clustering using biomarkers and s-Tf. Left: Dendrogram (yellow = MCI; red = AD; green = CT) and distribution of patients within the four clusters. Right: Heatmap using the median value of the features (Z-score unit) in each cluster. AD, Alzheimer's Disease; CT, neurological control; MCI, Mild Cognitive Impairment.

Considering the subpopulation including data of s-Tf (*N* = 29 patients), the results for HAC using same feature sets as in sections SBs and SBs + Iron in CSF are reported in Supplementary Figures 2, 3. In this subpopulation, the application of HAC using the feature set comprising biomarkers and s-Tf revealed four clusters (sizes: *N* = 7, *N* = 6, *N* = 9, *N* = 7), reported in Figure 4. Significant differences among clusters were found for all the features values between cluster 1 and 2 (for Aβ42, p-Tau *p* < 0.001; for s-Tf *p* = 0.02), for the p-Tau values (*p* < 0.001) when comparing clusters 1 and 3 and 1 and 4; s-Tf differed between clusters 2 and 4 (*p* = 0.001) and 3 and 4 (*p* < 0.001); Aβ42 was significantly different when comparing clusters 2 and 3 (*p* < 0.001) while the difference in Aβ42 between clusters 3 and 4 is borderline (*p* = 0.05).

Values of MMSE have been calculated for each cluster [cluster 1 = (22.6 ± 4.8); cluster 2 = (21.1 ± 3.6); cluster 3 = (21.5 ± 6.9); cluster 4 = (27.5 ± 2.8)].

In this case, clustering scores showed the following values: V-measure (0.32), ARI (0.18), AMI (0.25).

#### SBs + s-Tf + Iron in CSF

**Figure 5:**
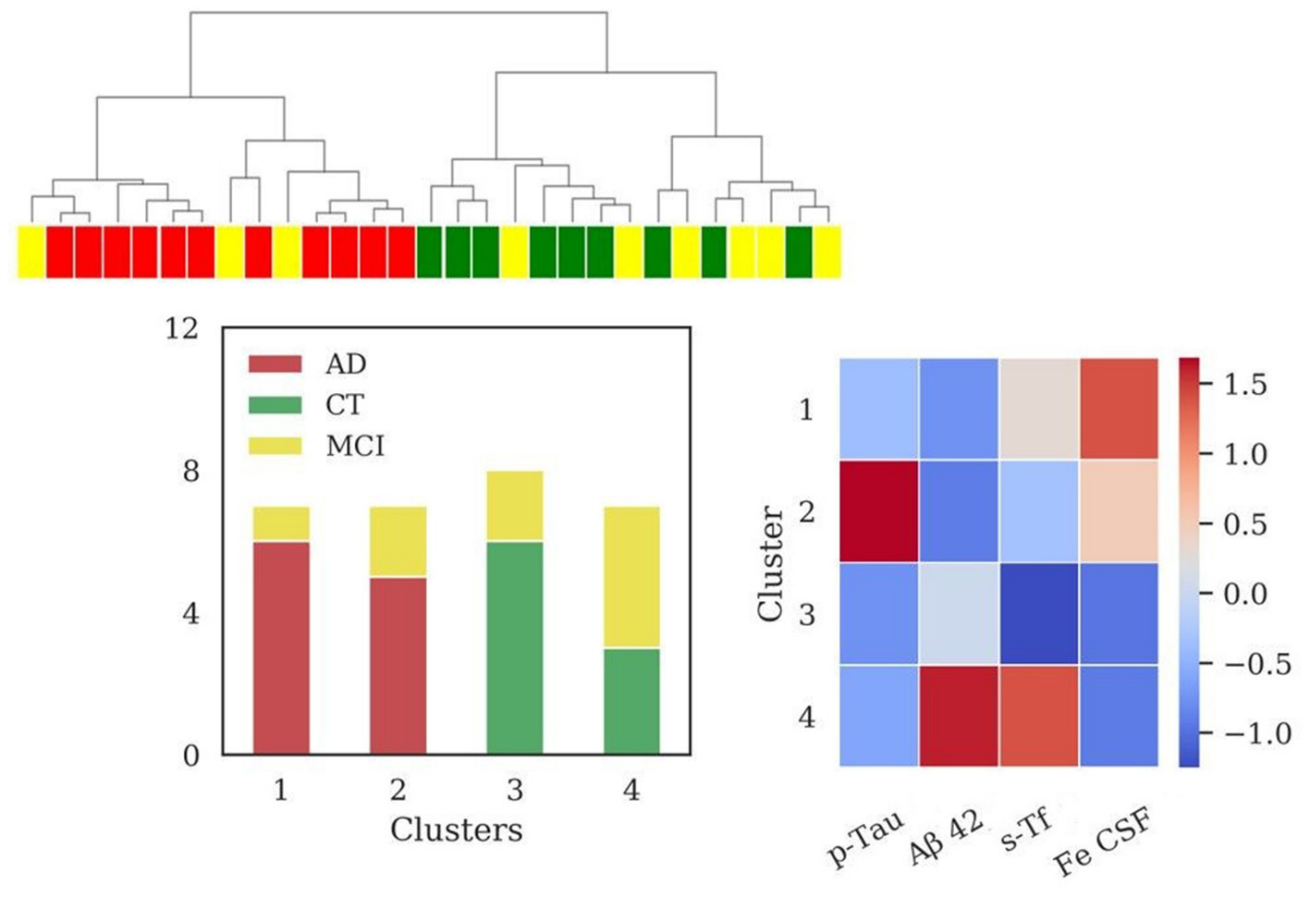
Results of hierarchical clustering using biomarkers, s-Tf and CSF iron. Left: Dendrogram (yellow = MCI; red = AD; green = CT) and distribution of patients within the four clusters. Right: Heatmap using the median value of the features (Z-score unit) in each cluster. AD, Alzheimer's Disease; CT, neurological control; MCI, Mild Cognitive Impairment.

Considering all the above features four clusters emerged (sizes: *N* = 7, *N* = 7, *N* = 8, *N* = 7). The application of HAC in the subpopulation for which s-Tf is available (Figure 5) reported an increase of clustering scores (V-measure = 0.43; ARI = 0.28; AMI = 0.37). Cluster 1 and cluster 2 are composed only by AD and MCI patients. One of these clusters (cluster 1) presented significant difference in the levels of s-Tf (*p* = 0.002), Iron CSF (*p* = 0.004), and p-Tau (*p* = 0.007) with respect to cluster 3 (mainly CT patients). Cluster 2 differed from cluster 4 (composed only by MCI and CT patients) in the biomarkers (for Aβ42 *p* = 0.004; for p-Tau *p* < 0.001) and s-Tf (*p* = 0.008) profiles. Clusters 1 and 2, as well as clusters 2 and 3, significantly differed only for p-Tau (*p* < 0.001). Finally, cluster 4 differed from cluster 3 only for the s-Tf values (p <0.001).

Values of MMSE for each cluster were: [cluster 1= (20.6 ± 6.9); cluster 2= (22.6 ± 4.8); cluster 3= (27.6 ± 2.5); cluster 4= (21.1 ± 3.6)].

The (Fe CSF/s-Tf) ratio in cluster 3 (0.15) and cluster 4 (0.11) is lower with respect to cluster 1 (0.23) and cluster 2 (0.24), in which the ratio is increased. Considering the relevant subpopulation, the (Fe CSF/s-Tf) ratio showed highest values for AD (0.24 ± 0.07), followed by MCI (0.18 ± 0.09) and finally CT group (0.13± 0.06), reporting a significant difference between AD and CT (*p* < 0.01).

We performed additional HAC analysis considering age correction for the variable s-Tf, showing a sharper separation on s-Tf profiles albeit without substantial differences in the cluster composition (Supplementary Figures 4–6).

### Classification Models

We finally used a Linear SVM and LR model to evaluate the classification performance based on the same feature sets used for the clustering analysis in the population (*N* = 47). For SVM, the classification performance (AUROC) using SBs (Aβ42, p-Tau) and SBs + Iron in CSF was (0.74 ± 0.14) and (0.73 ± 0.12), respectively. In the first case, the values of feature importance for the biomarkers set showed a higher weight for Aβ42 (0.21 ± 0.18) respect to p-Tau (0.15 ± 0.11). In the second case, CSF iron reported a higher value (0.07 ± 0.09) respect to p-Tau (0.06 ± 0.09) and Aβ42 (0.03 ± 0.12). For LR model, AUROC using SBs and SBs + Iron in CSF was (0.77 ± 0.12) and (0.75 ± 0.12), respectively. The values of feature importance for the biomarkers set showed a higher weight for Aβ42 (0.20 ± 0.14) respect to p-Tau (0.11 ± 0.13). Even in LR model, CSF iron reported a higher value (0.10 ± 0.02) respect to p-Tau (0.06 ± 0.13) and Aβ42 (0.09 ± 0.08). In our dataset, for both models the addition of age as feature did not improve the AUROC, and the value of feature importance for age was not relevant (around zero).

## Discussion

The present results support the hypothesis that iron accumulation is involved in AD neurodegeneration. In clinical practice, the pathological changes occurring in AD can be detected by the use of biomarkers in different modalities, among which the evaluation on CSF (i.e., Tau and Aβ biomarkers) is less accessible but presents lower intrinsic protease activity than blood and reflects brain changes, helping to diagnose AD pathology in both the prodromal and the dementia stages (Lashley et al., 2018).

In our study, both the “standard” biomarkers significantly differed between AD and CT groups (only CSF Aβ42 between AD and MCI), but this result requires confirmation in a larger cohort of patients also due to the large dispersion of biomarker values in our MCI population (Table 1). In fact, core-AD hallmarks are present also in elder healthy people with good cognitive function (Driscoll and Troncoso, 2011), probably showing different patterns only with respect to the AD brain. We added the information on iron content in CSF, intending to discover shared profiles and potentially improve early diagnosis. The analytical measurements of total iron in CSF by means of GF-AAS is expected to provide an accurate quantification, reflecting the iron status in brain patients more directly than brain imaging techniques. Indeed, biochemical changes in the brain, occurring in preclinical phases produce corresponding alterations in the CSF (Jack et al., 2010). Iron concentration in CSF is minimal and therefore very difficult to measure, requiring accurate and highly sensitive techniques such as atomic absorption spectrometry, providing reproducible and reliable results, without the need for hard pre-treatment of CSF samples. Moreover, this measurement is not currently used in clinical practice, leading an added value for the standard information on iron status in the brain.

Several studies evaluated iron levels in biological fluids, and meta-analysis conducted by Tao et al. showed lower iron in serum and an iron overload in several specific brain regions of AD patients, however highlighting the need for further studies to evaluate iron in CSF (Tao et al., 2014).

In the present work, CSF iron concentration in 69 patients was analyzed, finding a statistically significant increase in the total iron concentration in AD with respect to CT patients, and between AD and MCI (Figure 1), showing a potential discriminating power of our analysis. Iron dysregulation could generate a progressively toxic environment during the different stages of dementia. In fact, a well-known consequence of increased iron concentration is the production of ROS, leading to DNA damage and cell death (Zecca et al., 2004), that feature AD.

The concentration of iron in CSF is very low, and it has been suggested that Tf saturation in the CSF is much higher than in the periphery and that a larger proportion of free iron circulates the CNS (Leitner and Connor, 2012). An imbalance of free iron can be responsible for toxic damage taking part in Fenton reaction and consequently to the onset of neurodegeneration. Interestingly, it is reported that different stages of cognitive and functional impairment are associated with changes in CSF reactive iron, possibly in relation to the development of cognitive and functional decline (Lavados et al., 2008).

The importance of iron in AD and aging has been shown also from the evaluation of altered local levels of proteins regulating iron levels, such as transferrin (Loeffler et al., 1995; Lu et al., 2018).

Then, it has been assessed that CSF ferritin, the iron storage protein of the body, plays a role in AD. It has been demonstrated that CSF ferritin, assumed to be an index of brain iron load, is strongly associated with CSF apolipoprotein E levels and was elevated by the Alzheimer's risk allele, APOE-ε4 (Ayton et al., 2015). Also, CSF ferritin levels have been associated with longitudinal changes in CSF Aβ and Tau, showing that iron might facilitate Aβ deposition in AD and accelerate the disease process (Ayton et al., 2018). These evidences provide proofs that a disturbance in iron metabolism can be involved in neurodegenerative processes.

To check whether our technique was accurate enough to detect small differences in the iron concentration in CSF and to evaluate the possibility to discriminate between AD and other dementias, in the first part of our study we compared AD and FTD patients. The results (Figure 1) showed a marked difference in iron concentrations in FTD patients with respect to AD, suggesting a possible different role for this metal in these two types of dementia. This result should be confirmed in larger cohorts, however, different levels of biological metals in CSF have been showed in different neurodegenerative diseases (Hozumi et al., 2011), in particular AD, Amyotrophic Lateral Sclerosis, and Parkinson disease, so it is plausible that a difference exists also in FTD (Ashraf and So, 2020).

AD is a complex disease (Devi and Scheltens, 2018), requiring advanced computational algorithms to discover deep relationships in the data and their relative patterns. In this work we applied clustering analysis, a powerful technique suitable to discover patterns and similar subgroups, which has been successfully applied in recent studies on AD (Racine et al., 2016; Alashwal et al., 2019; Toschi et al., 2019). We tested how considering different sets of features can better diagnose the progression of AD and point out new potential pathological mechanisms involved in neurodegeneration. Results from hierarchical clustering analysis revealed that using the standard AD biomarkers Aβ42 and p-Tau, two groups of patients presented alternative signatures (clusters 1 and 2, Figure 2). One of these (cluster 1, Figure 2) could be associated with the AD profile showing low levels of Aβ42 and high levels of p-Tau, reflecting the accumulation of amyloid plaques and neurofibrillary tangles. However, a third subgroup emerged (cluster 3, Figure 2) with a heterogeneous composition of AD, MCI, and CT, underlining that the two standard biomarkers alone are unable of a sharp discrimination of the patient status. Probably, the sparse presence of MCI patients in all the clusters is due to the wide spectrum of cognitive and functional impairment that is captured by the MCI designation, impacting the heterogeneity of outcomes (Roberts and Knopman, 2013).

Interestingly, the information on iron concentration in CSF added one more cluster, generating two separated groups composed of patients classified with diagnosis of AD and MCI (clusters 3 and 4, Figure 3), both presenting low values of MMSE. These clusters differed for p-Tau and CSF iron levels, albeit presenting similar Aβ42 profile. Cluster 3 could be considered as a typical AD profile, with low levels of Aβ42 and high values for p-Tau, and in addition higher values of iron with respect to the two clusters containing mainly CT patients (clusters 1 and 2, Figure 3). Cluster 4 presents low levels of Aβ42, lower levels of p-Tau with respect to cluster 3, and the highest levels of CSF iron. The improvement of clustering scores assessed a better discrimination of patients by adding the iron content in CSF to the standard biomarkers. One possible interpretation of these results is that cluster 4 could be associated to patients in stages of dementia in which p-Tau starts to aggregate but deposition of Aβ plaques is already present.

In fact, according to the current models describing the timing of pathophysiological brain events in relation to the clinical course, preclinical phases of AD are characterized by plaques deposition, followed by later spread of neurofibrillary tangles (Jack et al., 2010; Long and Holtzman, 2019). Furthermore, the higher iron concentration combined with lower levels of p-Tau could suggest a harmful interaction between iron and p-Tau accumulation at early stages, inducing or worsening neurodegenerative events. This hypothesis is consistent with several studies, supporting the evidence that iron can promote the aggregation and pathogenicity of Tau (Smith et al., 1997; Yamamoto et al., 2002; Lovell et al., 2004; Ahmadi et al., 2017; Spotorno et al., 2020). Chelation therapies, based on intranasal deferoxamine treatment, may exert suppressive effects on the iron-induced tau phosphorylation, providing a valuable approach in preventing AD progression (Guo et al., 2013). In addition, further studies on patients reported an investigation of novel treatment strategy based on a metal-protein-attenuating compound to reduce toxic properties of Aβ mediated by copper and zinc (Ritchie et al., 2003; Lannfelt et al., 2008).

According to our results, the clusters containing patients affected by AD showed an increased level of iron with low levels of Aβ42, hallmark for senile plaques deposition, which confirms the link between iron and Aβ plaques. There are evidences for a variety of interactions between iron and Aβ: iron accumulates and co-localizes with Aβ plaques (Connor et al., 1992; Meadowcroft et al., 2009; Ndayisaba et al., 2019), their binding can form redox reactive and toxic species (Nakamura et al., 2007; Bousejra-ElGarah et al., 2011), with evidence for the formation of an iron-amyloid complex (Telling et al., 2017), and also iron levels can increase prior to plaques formation in an animal model of AD (Leskovjan et al., 2011). Increased iron levels are believed to enhance Aβ production via the downregulation of furin (Silvestri and Camaschella, 2008) and the iron regulatory pathways are also involved in proteostasis of Amyloid Precursor Protein (APP) (Rogers et al., 2008; Duce et al., 2010). It has been hypothesized that brain oxidative damage concurs to AD pathogenesis before Aβ accumulation (Praticò et al., 2001), therefore iron accumulation might precede and cause the formation of plaques. Recently it has been proposed that an increase in the intracellular labile iron levels, due to mitochondria dysfunction, enhances the rate of APP synthesis and the activity of APP cleavage by beta-secretase resulting in Aβ formation (Kozlov et al., 2017). In addition, a recent study confirmed the link between iron retention in cells and mislocalization of APP, due to alteration of ferroportin activity in the modulation of iron efflux: this effect causes a change in endocytic trafficking with consequent neuronal iron elevation and oxidative damage that feature AD pathology (Tsatsanis et al., 2020).

Further connections between iron and lipoprotein metabolism have been detected, highlighting causative interaction and synergies between genes of iron homeostasis and established genetic risk factors of AD, such as APOE4, suggesting the iron metabolism as a possible therapeutic target (Tisato et al., 2018).

Emerging evidence suggested that blood iron homeostasis is altered in AD and already in MCI (Faux et al., 2014; Ashraf et al., 2020; Guan et al., 2020), including systemic variation of markers of iron metabolism, such as transferrin saturation and ceruloplasmin/transferrin ratio in serum (Squitti et al., 2010). Interestingly, in our case, we found a negative correlation between t-Tau and Tf in serum in AD that can support a role of Tf in neurodegeneration. Despite our observations require further confirmation in a larger cohort of patients, cluster analysis also found different concentrations of Tf in serum and iron levels in CSF for patients with MCI and dementia.

Using features set including standard biomarkers and s-Tf, four clusters emerged, with different profiles of s-Tf. Cluster 1 (Figure 4) containing mainly AD patients, associated typical AD profile with a lower level of s-Tf compared to two of three remaining clusters. This result might support that serum iron is lower in AD than in healthy controls (Tao et al., 2014), and that decreased plasmatic iron in AD could be due to transferrin desaturation (Hare et al., 2015), pointing out a role for systemic variations of iron metabolism in neurodegeneration. However, the other cluster comprising AD and MCI patients (cluster 3, Figure 4) reported a higher level of s-Tf with respect to the cluster 4 (Figure 4) composed of MCI and CT patients, requiring a further investigation on the s-Tf profiles in a larger population. Using a step-by-step approach, we finally used the full set of features, including also information on iron concentration in CSF, which largely improved the discrimination of patients according to their clinical diagnosis. Clustering analysis unraveled four clusters, visualized in the dendrogram (Figure 5), in which AD and CT patients are well-separated, supported by a good improvement of clustering scores. As previously discussed, the presence of MCI patients in all clusters reflected their wide spectrum in the current MCI diagnosis. Interestingly, a recent study reported that postmortem MRI and histology demonstrated differential iron accumulation in early- and late-onset AD (Bulk et al., 2018), showing the presence of various distribution patterns for iron accumulation in subtypes of AD patients.

Two subgroups containing AD and MCI patients (clusters 1 and 2, Figure 5) differed in p-Tau levels and showed CSF iron levels higher with respect to the clusters composed of CT and MCI (clusters 3 and 4, Figure 5). This result sustains our previous hypothesis that iron concentration and p-Tau levels in CSF could play a crucial role in differentiating the actual stage of dementia.

The interpretation of clusters 3 and 4 (Figure 5) is less simple. The different profiles of s-Tf for these clusters, containing CT and MCI patients, could be due both to the small size of samples requiring further investigation in a larger cohort.

Significant differences in s-Tf profiles have been detected in the clusters containing mainly AD and CT, respectively, showing also an increased ratio between the iron content in CSF and s-Tf in clusters only formed by patients affected by dementia and for the AD with respect to CT group. It is increasingly recognized that AD is a clinically heterogeneous disease with multiple causes, with an important role for brain vasculature (Montagne et al., 2017; Sweeney et al., 2018a). Our approach could be considered an indirect evaluation of the potential connection between iron homeostasis and blood-brain barrier (BBB) dysfunction. BBB dysfunction is a mechanism involved in the neurodegeneration and subsequently in cognitive impairment (Nation et al., 2019), and recently included in a hypothetical model of AD biomarkers (Sweeney et al., 2018b). Interestingly, the export mechanisms at the BBB level are altered in dementia, leading to potential targets for treatment (Pahnke et al., 2014). AD is characterized by altered BBB permeability and a link between iron-overload and BBB breakdown and brain mitochondrial dysfunction has been demonstrated (Sripetchwandee et al., 2016).

In fact, BBB prevents the diffusion of Tf from blood into CNS, as well as the migration of non-transferrin bound iron (NTBI), potentially toxic from the brain. Tf is transported across brain capillary endothelial cells following an endocytic mechanism mediated by Tf-receptors (Moos et al., 2007). The breakdown or alteration of this system could be part of the cause for pathological accumulation of iron into the brain and consequently in the CSF. In fact, CSF can be produced both via the choroid plexus or by the interstitial fluid of the brain (Nakada and Kwee, 2019).

Moreover, various studies proposed that iron trafficking across the blood-brain capillaries is involved in the aggregation of Aβ peptides leading to the potential onset of AD (McCarthy and Kosman, 2015), and also that iron accumulation may be associated with the age-induced changes in the expression of iron metabolism proteins in the brain (Lu et al., 2017).

To extract further information on the data, useful for future clinical studies, machine learning models have been trained to evaluate the impact of the inclusion of iron-related data in the diagnostic power of dementia. We selected LR model and linear SVM, one of the most used techniques for AD classification problem (Tanveer et al., 2020), giving good generalization performances also for small samples. In particular, the present results for both models were comparable, underlining a potential relevance of the iron-related feature (CSF iron) for the classification of AD, CT, MCI patients. Although AUROC did not detect a significant increase, in ranking the relevance of features the addition of CSF iron could suggest a potential role in the improvement of the diagnostic power of AD, MCI, and CT patients, provided further investigation on larger samples are performed. Moreover, the cross-sectional nature of our study is a limitation of the present investigation and longitudinal studies will be necessary to clarify the involvement of abnormal iron homeostasis in different stages of the disease process, i.e., including iron-related data from blood or MRI. Additionally, further studies with larger samples will be useful to quantitatively parse out our results and to confirm the stratification of patients turned out in the cluster analysis.

Finally, the use of cluster analysis proved its potential utility for identifying patterns of variables that might characterize disease progression. The addition of iron-related data to the core-biomarkers can help to capture the multifaceted nature of AD, co-characterized by Aβ plaques deposition and neurofibrillary tangles aggregation as well as other related processes. Future works should focus on the evaluation of abnormal iron concentrations in different stages of dementia, which can generate deposition in CSF or changes in circulating iron (protein-bound or free) arising from possible imbalances of the blood-brain exchange of iron. The evaluation of iron in the CSF can improve the tuning of personalized therapeutic strategies based on systemic or intrathecal administration of chelating agents acting directly into the brain.

In conclusion, our results support the evidence of iron overload in AD, and consequently the hypothesis that different clinical conditions with specific backgrounds involve the actual iron brain levels. Cluster analysis revealed a new potential stratification of patients when new parameters, related to the iron concentration in serum and CSF, are accounted for, advancing our understanding of the role of iron dysregulation in AD pathophysiology. Tight regulation of iron metabolism is pivotal to warrant neuronal homeostasis and its investigation can prompt avenues for both research on new pathological mechanisms involved in neurodegeneration and development of new treatments. The potential addition of iron-related data in clinical evaluation of dementia could improve the early diagnostic power and support new personalized disease-modifying therapies based on iron chelation able to slow the progression and worsening of the neurodegenerative processes.

## Data Availability Statement

The raw data supporting the conclusions of this article will be made available by the authors, without undue reservation.

## Ethics Statement

The studies involving human participants were reviewed and approved by Comitato Etico Interaziendale A.O.U. Città della Salute e della Scienza di Torino - A.S.L. Città di Torino. The patients/participants provided their written informed consent to participate in this study.

## Author Contributions

EF, SB, FD'A, CG, OA, and IR: conceptualization and design. EF, SA, and OA: methodology. PC, GDF, AI, and IR: patient enrollment and clinical data managing. EF: computational design and manuscript preparation. SB, FD'A, CG, OA, PC, GDF, and IR: writing-review and supervision. All authors: contributed to the article and approved the submitted version.

## Conflict of Interest

The authors declare that the research was conducted in the absence of any commercial or financial relationships that could be construed as a potential conflict of interest.

## References

[B1] Ahmadi S. Ebralidze I. I. She Z. Kraatz H.-B. (2017). Electrochemical studies of tau protein-iron interactions—potential implications for Alzheimer's Disease. Electrochim. Acta 236, 384–393. 10.1016/j.electacta.2017.03.175

[B2] Aizenstein H. J. Nebes R. D. Saxton J. A. Price J. C. Mathis C. A. Tsopelas N. D.. (2008). Frequent amyloid deposition without significant cognitive impairment among the elderly. Arch. Neurol. 65, 1509–1517. 10.1001/archneur.65.11.1509 19001171 PMC2636844

[B3] Alashwal H. El Halaby M. Crouse J. J. Abdalla A. Moustafa A. A. (2019). The application of unsupervised clustering methods to Alzheimer's disease. Front. Comput. Neurosci. 13:31. 10.3389/fncom.2019.00031 31178711 PMC6543980

[B4] Albert M. S. DeKosky S. T. Dickson D. Dubois B. Feldman H. H. Fox N. C.. (2011). The diagnosis of mild cognitive impairment due to Alzheimer's disease: recommendations from the National Institute on Aging-Alzheimer's Association workgroups on diagnostic guidelines for Alzheimer's disease. Alzheimers. Dement. 7, 270–279. 10.1016/j.jalz.2011.03.008 21514249 PMC3312027

[B5] Ashraf A. Ashton N. J. Chatterjee P. Goozee K. Shen K. Fripp J.. (2020). Plasma transferrin and hemopexin are associated with altered Aβ uptake and cognitive decline in Alzheimer's disease pathology. Alzheimer's Res. Ther. 12:72. 10.1186/s13195-020-00634-1 32517787 PMC7285604

[B6] Ashraf A. So P.-W. (2020). Spotlight on ferroptosis: iron-dependent cell death in Alzheimer's disease. Front. Aging Neurosci. 12:196. 10.3389/fnagi.2020.00196 32760266 PMC7371849

[B7] Ayton S. Diouf I. Bush A. I. Alzheimer's disease Neuroimaging Initiative (2018). Evidence that iron accelerates Alzheimer's pathology: a CSF biomarker study. J. Neurol. Neurosurg. Psychiatr. 89, 456–460. 10.1136/jnnp-2017-316551 28939683

[B8] Ayton S. Faux N. G. Bush A. I. (2015). Ferritin levels in the cerebrospinal fluid predict Alzheimer's disease outcomes and are regulated by APOE. Nat. Commun. 6:6760. 10.1038/ncomms7760 25988319 PMC4479012

[B9] Ayton S. Wang Y. Diouf I. Schneider J. A. Brockman J. Morris M. C.. (2019). Brain iron is associated with accelerated cognitive decline in people with Alzheimer pathology. Mol. Psychiatry 25, 2932–2941. 10.1038/s41380-019-0375-7 30778133 PMC6698435

[B10] Bousejra-ElGarah F. Bijani C. Coppel Y. Faller P. Hureau C. (2011). Iron(II) binding to amyloid-β, the Alzheimer's peptide. Inorg. Chem. 50, 9024–9030. 10.1021/ic201233b 21800824

[B11] Bulk M. Abdelmoula W. M. Nabuurs R. J. A. van der Graaf L. M. Mulders C. W. H. Mulder A. A.. (2018). Postmortem MRI and histology demonstrate differential iron accumulation and cortical myelin organization in early- and late-onset Alzheimer's disease. Neurobiol. Aging 62, 231–242. 10.1016/j.neurobiolaging.2017.10.017 29195086

[B12] Cicero C. E. Mostile G. Vasta R. Rapisarda V. Signorelli S. S. Ferrante M.. (2017). Metals and neurodegenerative diseases. a systematic review. Environ. Res. 159, 82–94. 10.1016/j.envres.2017.07.048 28777965

[B13] Connor J. R. Snyder B. S. Beard J. L. Fine R. E. Mufson E. J. (1992). Regional distribution of iron and iron-regulatory proteins in the brain in aging and Alzheimer's disease. J. Neurosci. Res. 31, 327–335. 10.1002/jnr.490310214 1573683

[B14] Cortes C. Vapnik V. (1995). Support-vector networks. Mach. Learn. 20, 273–297. 10.1007/BF00994018

[B15] Devi G. Scheltens P. (2018). Heterogeneity of Alzheimer's disease: consequence for drug trials? Alzheimer's Res. Ther. 10:122. 10.1186/s13195-018-0455-y 30567585 PMC6300886

[B16] Di Fede G. Catania M. Maderna E. Ghidoni R. Benussi L. Tonoli E.. (2018). Molecular subtypes of Alzheimer's disease. Sci. Rep. 8:3269. 10.1038/s41598-018-21641-1 29459625 PMC5818536

[B17] Dixon S. J. Lemberg K. M. Lamprecht M. R. Skouta R. Zaitsev E. M. Gleason C. E.. (2012). Ferroptosis: an iron-dependent form of nonapoptotic cell death. Cell 149, 1060–1072. 10.1016/j.cell.2012.03.042 22632970 PMC3367386

[B18] Dormann C. F. Elith J. Bacher S. Buchmann C. Carl G. Carré G.. (2013). Collinearity: a review of methods to deal with it and a simulation study evaluating their performance. Ecography 36, 27–46. 10.1111/j.1600-0587.2012.07348.x

[B19] Driscoll I. Troncoso J. (2011). Asymptomatic Alzheimer's disease: a prodrome or a state of resilience? Curr. Alzheimer Res. 8, 330–335. 10.2174/156720511795745348 21222594 PMC3286868

[B20] Dubois B. Feldman H. H. Jacova C. Cummings J. L. Dekosky S. T. Barberger-Gateau P.. (2010). Revising the definition of Alzheimer's disease: a new lexicon. Lancet Neurol. 9, 1118–1127. 10.1016/S1474-4422(10)70223-4 20934914

[B21] Dubois B. Feldman H. H. Jacova C. Dekosky S. T. Barberger-Gateau P. Cummings J.. (2007). Research criteria for the diagnosis of Alzheimer's disease: revising the NINCDS-ADRDA criteria. Lancet Neurol. 6, 734–746. 10.1016/S1474-4422(07)70178-3 17616482

[B22] Duce J. A. Bush A. I. (2010). Biological metals and Alzheimer's disease: implications for therapeutics and diagnostics. Prog. Neurobiol. 92, 1–18. 10.1016/j.pneurobio.2010.04.003 20444428

[B23] Duce J. A. Tsatsanis A. Cater M. A. James S. A. Robb E. Wikhe K.. (2010). Iron-export ferroxidase activity of β-amyloid precursor protein is inhibited by zinc in Alzheimer's disease. Cell 142, 857–867. 10.1016/j.cell.2010.08.014 20817278 PMC2943017

[B24] Eid R. Arab N. T. T. Greenwood M. T. (2017). Iron mediated toxicity and programmed cell death: A review and a re-examination of existing paradigms. Biochim. Biophys. Acta Mol. Cell. Res. 1864, 399–430. 10.1016/j.bbamcr.2016.12.002 27939167

[B25] Fagan A. M. Roe C. M. Xiong C. Mintun M. A. Morris J. C. Holtzman D. M. (2007). Cerebrospinal fluid tau/beta-amyloid(42) ratio as a prediction of cognitive decline in nondemented older adults. Arch. Neurol. 64, 343–349. 10.1001/archneur.64.3.noc60123 17210801

[B26] Faux N. G. Rembach A. Wiley J. Ellis K. A. Ames D. Fowler C. J.. (2014). An anemia of Alzheimer's disease. Mol. Psychiatry 19, 1227–1234. 10.1038/mp.2013.178 24419041

[B27] Fiandaca M. S. Mapstone M. E. Cheema A. K. Federoff H. J. (2014). The critical need for defining preclinical biomarkers in Alzheimer's disease. Alzheimers. Dement. 10, S196–212. 10.1016/j.jalz.2014.04.015 24924671

[B28] Gozzelino R. Arosio P. (2016). Iron homeostasis in health and disease. Int. J. Mol. Sci. 17:130. 10.3390/ijms17010130 PMC4730371 26805813

[B29] Guan J. Wang P. Lu L. Zhao G. (2020). Association of plasma transferrin with cognitive decline in patients with mild cognitive impairment and Alzheimer's disease. Front. Aging Neurosci. 12:38. 10.3389/fnagi.2020.00038 32226377 PMC7080847

[B30] Guo C. Wang P. Zhong M.-L. Wang T. Huang X.-S. Li J.-Y.. (2013). Deferoxamine inhibits iron induced hippocampal tau phosphorylation in the Alzheimer transgenic mouse brain. Neurochem. Int. 62, 165–172. 10.1016/j.neuint.2012.12.005 23262393

[B31] Hao S. Liang B. Huang Q. Dong S. Wu Z. He W.. (2018). Metabolic networks in ferroptosis. Oncol. Lett. 15, 5405–5411. 10.3892/ol.2018.8066 29556292 PMC5844144

[B32] Hare D. Ayton S. Bush A. Lei P. (2013). A delicate balance: Iron metabolism and diseases of the brain. Front. Aging Neurosci. 5:34. 10.3389/fnagi.2013.00034 23874300 PMC3715022

[B33] Hare D. J. Doecke J. D. Faux N. G. Rembach A. Volitakis I. Fowler C. J.. (2015). Decreased plasma iron in Alzheimer's disease is due to transferrin desaturation. ACS Chem. Neurosci. 6, 398–402. 10.1021/cn5003557 25588002

[B34] Henry M. S. Passmore A. P. Todd S. McGuinness B. Craig D. Johnston J. A. (2013). The development of effective biomarkers for Alzheimer's disease: a review. Int. J. Geriatr. Psychiatry 28, 331–340. 10.1002/gps.3829 22674539

[B35] Hozumi I. Hasegawa T. Honda A. Ozawa K. Hayashi Y. Hashimoto K.. (2011). Patterns of levels of biological metals in CSF differ among neurodegenerative diseases. J. Neurol. Sci. 303, 95–99. 10.1016/j.jns.2011.01.003 21292280

[B36] International A. D. (2019). World Alzheimer Report 2019: Attitudes to dementia | Alzheimer's Disease International. Available online at: https://www.alz.co.uk/research/world-report-2019 (accessed August 27, 2020).

[B37] Ittner L. M. Götz J. (2011). Amyloid-β and tau — a toxic pas de deux in Alzheimer's disease. Nat. Rev. Neurosci. 12, 67–72. 10.1038/nrn2967 21193853

[B38] Jack C. R. Knopman D. S. Jagust W. J. Shaw L. M. Aisen P. S. Weiner M. W.. (2010). Hypothetical model of dynamic biomarkers of the Alzheimer's pathological cascade. Lancet Neurol. 9, 119–128. 10.1016/S1474-4422(09)70299-6 20083042 PMC2819840

[B39] Kozlov S. Afonin A. Evsyukov I. Bondarenko A. (2017). Alzheimer's disease: as it was in the beginning. Rev. Neurosci. 28, 825–843. 10.1515/revneuro-2017-0006 28704198

[B40] Lane D. J. R. Ayton S. Bush A. I. (2018). Iron and Alzheimer's disease: an update on emerging mechanisms. J. Alzheimers. Dis. 64, S379–S395. 10.3233/JAD-179944 29865061

[B41] Lannfelt L. Blennow K. Zetterberg H. Batsman S. Ames D. Harrison J.. (2008). Safety, efficacy, and biomarker findings of PBT2 in targeting Abeta as a modifying therapy for Alzheimer's disease: a phase IIa, double-blind, randomised, placebo-controlled trial. Lancet Neurol. 7, 779–786. 10.1016/S1474-4422(08)70167-4 18672400

[B42] Lashley T. Schott J. M. Weston P. Murray C. E. Wellington H. Keshavan A.. (2018). Molecular biomarkers of Alzheimer's disease: progress and prospects. Dis. Model. Mech. 11:dmm031781. 10.1242/dmm.031781 29739861 PMC5992610

[B43] Lavados M. Guillón M. Mujica M. C. Rojo L. E. Fuentes P. Maccioni R. B. (2008). Mild cognitive impairment and Alzheimer patients display different levels of redox-active CSF iron. J. Alzheimers. Dis. 13, 225–232. 10.3233/JAD-2008-13211 18376063

[B44] Leitner D. F. Connor J. R. (2012). Functional roles of transferrin in the brain. Biochim. Biophys. Acta 1820, 393–402. 10.1016/j.bbagen.2011.10.016 22138408

[B45] Leskovjan A. C. Kretlow A. Lanzirotti A. Barrea R. Vogt S. Miller L. M. (2011). Increased brain iron coincides with early plaque formation in a mouse model of Alzheimer's disease. Neuroimage 55, 32–38. 10.1016/j.neuroimage.2010.11.073 21126592 PMC3031776

[B46] Liu J.-L. Fan Y.-G. Yang Z.-S. Wang Z.-Y. Guo C. (2018). Iron and Alzheimer's disease: from pathogenesis to therapeutic implications. Front. Neurosci. 12:632. 10.3389/fnins.2018.00632 30250423 PMC6139360

[B47] Loeffler D. A. Connor J. R. Juneau P. L. Snyder B. S. Kanaley L. DeMaggio A. J.. (1995). Transferrin and iron in normal, Alzheimer's disease, and Parkinson's disease brain regions. J. Neurochem. 65, 710–724. 10.1046/j.1471-4159.1995.65020710.x 7616227

[B48] Long J. M. Holtzman D. M. (2019). Alzheimer disease: an update on pathobiology and treatment strategies. Cell 179, 312–339. 10.1016/j.cell.2019.09.001 31564456 PMC6778042

[B49] Lovell M. A. Xiong S. Xie C. Davies P. Markesbery W. R. (2004). Induction of hyperphosphorylated tau in primary rat cortical neuron cultures mediated by oxidative stress and glycogen synthase kinase-3. J. Alzheimers Dis. 6, 659–671; discussion 673-681. 10.3233/JAD-2004-6610 15665406

[B50] Lu C.-D. Ma J.-K. Luo Z.-Y. Tai Q.-X. Wang P. Guan P.-P. (2018). Transferrin is responsible for mediating the effects of iron ions on the regulation of anterior pharynx-defective-1α/β and Presenilin 1 expression via PGE2 and PGD2 at the early stage of Alzheimer's Disease. Aging 10, 3117–3135. 10.18632/aging.101615 30383537 PMC6286844

[B51] Lu L.-N. Qian Z.-M. Wu K.-C. Yung W.-H. Ke Y. (2017). Expression of iron transporters and pathological hallmarks of Parkinson's and Alzheimer's diseases in the brain of young, adult, and aged rats. Mol. Neurobiol. 54, 5213–5224. 10.1007/s12035-016-0067-0 27578012

[B52] McCarthy R. C. Kosman D. J. (2015). Iron transport across the blood-brain barrier: development, neurovascular regulation and cerebral amyloid angiopathy. Cell. Mol. Life Sci. 72, 709–727. 10.1007/s00018-014-1771-4 25355056 PMC4312246

[B53] McKhann G. M. Knopman D. S. Chertkow H. Hyman B. T. Jack C. R. Kawas C. H.. (2011). The diagnosis of dementia due to Alzheimer's disease: recommendations from the National Institute on Aging-Alzheimer's Association workgroups on diagnostic guidelines for Alzheimer's disease. Alzheimers. Dement. 7, 263–269. 10.1016/j.jalz.2011.03.005 21514250 PMC3312024

[B54] Meadowcroft M. D. Connor J. R. Smith M. B. Yang Q. X. (2009). MRI and histological analysis of beta-amyloid plaques in both human Alzheimer's disease and APP/PS1 transgenic mice. J. Magn. Reson. Imaging 29, 997–1007. 10.1002/jmri.21731 19388095 PMC2723054

[B55] Molinuevo J. L. Ayton S. Batrla R. Bednar M. M. Bittner T. Cummings J.. (2018). Current state of Alzheimer's fluid biomarkers. Acta Neuropathol. 136, 821–853. 10.1007/s00401-018-1932-x 30488277 PMC6280827

[B56] Montagne A. Zhao Z. Zlokovic B. V. (2017). Alzheimer's disease: A matter of blood–brain barrier dysfunction? J. Exp. Med. 214, 3151–3169. 10.1084/jem.20171406 29061693 PMC5679168

[B57] Moos T. Rosengren Nielsen T. Skjørringe T. Morgan E. H. (2007). Iron trafficking inside the brain. J. Neurochem. 103, 1730–1740. 10.1111/j.1471-4159.2007.04976.x 17953660

[B58] Nakada T. Kwee I. L. (2019). Fluid dynamics inside the brain barrier: current concept of interstitial flow, glymphatic flow, and cerebrospinal fluid circulation in the brain. Neuroscientist 25, 155–166. 10.1177/1073858418775027 29799313 PMC6416706

[B59] Nakamura M. Shishido N. Nunomura A. Smith M. A. Perry G. Hayashi Y.. (2007). Three histidine residues of amyloid-beta peptide control the redox activity of copper and iron. Biochemistry 46, 12737–12743. 10.1021/bi701079z 17929832

[B60] Nation D. A. Sweeney M. D. Montagne A. Sagare A. P. D'Orazio L. M. Pachicano M.. (2019). Blood–brain barrier breakdown is an early biomarker of human cognitive dysfunction. Nat. Med. 25, 270–276. 10.1038/s41591-018-0297-y 30643288 PMC6367058

[B61] Ndayisaba A. Kaindlstorfer C. Wenning G. K. (2019). Iron in neurodegeneration - cause or consequence? Front. Neurosci. 13:180. 10.3389/fnins.2019.00180 30881284 PMC6405645

[B62] Olney N. T. Spina S. Miller B. L. (2017). Frontotemporal dementia. Neurol. Clin. 35, 339–374. 10.1016/j.ncl.2017.01.008 28410663 PMC5472209

[B63] Pahnke J. Langer O. Krohn M. (2014). Alzheimer's and ABC transporters–new opportunities for diagnostics and treatment. Neurobiol. Dis. 72, 54–60. 10.1016/j.nbd.2014.04.001 24746857 PMC4199932

[B64] Palmqvist S. Janelidze S. Quiroz Y. T. Zetterberg H. Lopera F. Stomrud E.. (2020). Discriminative accuracy of plasma Phospho-tau217 for Alzheimer disease vs other neurodegenerative disorders. JAMA 324, 772–781. 10.1001/jama.2020.12134 32722745 PMC7388060

[B65] Pantopoulos K. Porwal S. K. Tartakoff A. Devireddy L. (2012). Mechanisms of mammalian iron homeostasis. Biochemistry 51, 5705–5724. 10.1021/bi300752r 22703180 PMC3572738

[B66] Pedregosa F. Varoquaux G. Gramfort A. Michel V. Thirion B. Grisel O.. (2011). Scikit-learn: machine learning in python. J. Mach. Learn. Res. 12, 2825–2830.

[B67] Petersen R. C. (2004). Mild cognitive impairment as a diagnostic entity. J. Intern. Med. 256, 183–194. 10.1111/j.1365-2796.2004.01388.x 15324362

[B68] Petersen R. C. Knopman D. S. Boeve B. F. Geda Y. E. Ivnik R. J. Smith G. E.. (2009). Mild cognitive impairment: ten years later. Arch. Neurol. 66, 1447–1455. 10.1001/archneurol.2009.266 20008648 PMC3081688

[B69] Praticò D. Uryu K. Leight S. Trojanoswki J. Q. Lee V. M. (2001). Increased lipid peroxidation precedes amyloid plaque formation in an animal model of Alzheimer amyloidosis. J. Neurosci. 21, 4183–4187. 10.1523/JNEUROSCI.21-12-04183.2001 11404403 PMC6762743

[B70] Price J. L. McKeel D. W. Buckles V. D. Roe C. M. Xiong C. Grundman M.. (2009). Neuropathology of nondemented aging: presumptive evidence for preclinical Alzheimer disease. Neurobiol. Aging 30, 1026–1036. 10.1016/j.neurobiolaging.2009.04.002 19376612 PMC2737680

[B71] Rabinovici G. D. Carrillo M. C. Forman M. DeSanti S. Miller D. S. Kozauer N.. (2016). Multiple comorbid neuropathologies in the setting of Alzheimer's disease neuropathology and implications for drug development. Alzheimers Dement. 3, 83–91. 10.1016/j.trci.2016.09.002 29067320 PMC5651346

[B72] Racine A. M. Koscik R. L. Berman S. E. Nicholas C. R. Clark L. R. Okonkwo O. C.. (2016). Biomarker clusters are differentially associated with longitudinal cognitive decline in late midlife. Brain 139, 2261–2274. 10.1093/brain/aww142 27324877 PMC4958904

[B73] Rascovsky K. Hodges J. R. Knopman D. Mendez M. F. Kramer J. H. Neuhaus J.. (2011). Sensitivity of revised diagnostic criteria for the behavioural variant of frontotemporal dementia. Brain 134, 2456–2477. 10.1093/brain/awr179 21810890 PMC3170532

[B74] Ritchie C. W. Bush A. I. Mackinnon A. Macfarlane S. Mastwyk M. MacGregor L.. (2003). Metal-protein attenuation with iodochlorhydroxyquin (clioquinol) targeting Abeta amyloid deposition and toxicity in Alzheimer disease: a pilot phase 2 clinical trial. Arch. Neurol. 60, 1685–1691. 10.1001/archneur.60.12.1685 14676042

[B75] Roberts R. Knopman D. S. (2013). Classification and Epidemiology of MCI. Clin. Geriatr. Med. 29, 753–772. 10.1016/j.cger.2013.07.003 24094295 PMC3821397

[B76] Robinson J. L. Lee E. B. Xie S. X. Rennert L. Suh E. Bredenberg C.. (2018). Neurodegenerative disease concomitant proteinopathies are prevalent, age-related and APOE4-associated. Brain 141, 2181–2193. 10.1093/brain/awy146 29878075 PMC6022546

[B77] Rogers J. T. Bush A. I. Cho H.-H. Smith D. H. Thomson A. M. Friedlich A. L.. (2008). Iron and the translation of the amyloid precursor protein (APP) and ferritin mRNAs: riboregulation against neural oxidative damage in Alzheimer's disease. Biochem. Soc. Trans. 36, 1282–1287. 10.1042/BST0361282 19021541 PMC2746665

[B78] Schrag M. Mueller C. Oyoyo U. Smith M. A. Kirsch W. M. (2011). Iron, zinc and copper in the Alzheimer's disease brain: a quantitative meta-analysis. some insight on the influence of citation bias on scientific opinion. Prog. Neurobiol. 94, 296–306. 10.1016/j.pneurobio.2011.05.001 21600264 PMC3134620

[B79] Silvestri L. Camaschella C. (2008). A potential pathogenetic role of iron in Alzheimer's disease. J. Cell. Mol. Med. 12, 1548–1550. 10.1111/j.1582-4934.2008.00356.x 18466351 PMC3918070

[B80] Singh N. Haldar S. Tripathi A. K. Horback K. Wong J. Sharma D.. (2014). Brain iron homeostasis: from molecular mechanisms to clinical significance and therapeutic opportunities. Antioxid. Redox Signal. 20, 1324–1363. 10.1089/ars.2012.4931 23815406 PMC3935772

[B81] Smith M. A. Harris P. L. Sayre L. M. Perry G. (1997). Iron accumulation in Alzheimer disease is a source of redox-generated free radicals. Proc. Natl. Acad. Sci. U.S.A. 94, 9866–9868. 10.1073/pnas.94.18.9866 9275217 PMC23283

[B82] Sperling R. A. Aisen P. S. Beckett L. A. Bennett D. A. Craft S. Fagan A. M.. (2011). Toward defining the preclinical stages of Alzheimer's disease: Recommendations from the National Institute on Aging-Alzheimer's Association workgroups on diagnostic guidelines for Alzheimer's disease. Alzheimers. Dement. 7, 280–292. 10.1016/j.jalz.2011.03.003 21514248 PMC3220946

[B83] Spotorno N. Acosta-Cabronero J. Stomrud E. Lampinen B. Strandberg O. T. van Westen D.. (2020). Relationship between cortical iron and tau aggregation in Alzheimer's disease. Brain 143, 1341–1349. 10.1093/brain/awaa089 32330946 PMC7241946

[B84] Squitti R. Salustri C. Siotto M. Ventriglia M. Vernieri F. Lupoi D.. (2010). Ceruloplasmin/Transferrin ratio changes in Alzheimer's disease. Int. J. Alzheimers. Dis. 2011:231595. 10.4061/2011/231595 21234401 PMC3014694

[B85] Sripetchwandee J. Wongjaikam S. Krintratun W. Chattipakorn N. Chattipakorn S. C. (2016). A combination of an iron chelator with an antioxidant effectively diminishes the dendritic loss, tau-hyperphosphorylation, amyloids-β accumulation and brain mitochondrial dynamic disruption in rats with chronic iron-overload. Neuroscience 332, 191–202. 10.1016/j.neuroscience.2016.07.003 27403880

[B86] Sweeney M. D. Kisler K. Montagne A. Toga A. W. Zlokovic B. V. (2018a). The role of brain vasculature in neurodegenerative disorders. Nat. Neurosci. 21, 1318–1331. 10.1038/s41593-018-0234-x 30250261 PMC6198802

[B87] Sweeney M. D. Sagare A. P. Zlokovic B. V. (2018b). Blood–brain barrier breakdown in Alzheimer disease and other neurodegenerative disorders. Nat. Rev. Neurol. 14, 133–150. 10.1038/nrneurol.2017.188 29377008 PMC5829048

[B88] Tanveer M. Richhariya B. Khan R. U. Rashid A. H. Khanna P. Prasad M.. (2020). Machine learning techniques for the diagnosis of Alzheimer's disease: a review. ACM Trans. Multimedia Comput. Commun. Appl. 16, 30:1–30:35. 10.1145/3344998

[B89] Tao Y. Wang Y. Rogers J. T. Wang F. (2014). Perturbed iron distribution in Alzheimer's disease serum, cerebrospinal fluid, and selected brain regions: a systematic review and meta-analysis. J. Alzheimers. Dis. 42, 679–690. 10.3233/JAD-140396 24916541

[B90] Telling N. D. Everett J. Collingwood J. F. Dobson J. van der Laan G. Gallagher J. J.. (2017). Iron biochemistry is correlated with amyloid plaque morphology in an established mouse model of Alzheimer's disease. Cell Chem Biol. 24, 1205–1215.e3. 10.1016/j.chembiol.2017.07.014 28890316

[B91] Tisato V. Zuliani G. Vigliano M. Longo G. Franchini E. Secchiero P.. (2018). Gene-gene interactions among coding genes of iron-homeostasis proteins and APOE-alleles in cognitive impairment diseases. PLoS ONE 13:e0193867. 10.1371/journal.pone.0193867 29518107 PMC5843269

[B92] Toschi N. Lista S. Baldacci F. Cavedo E. Zetterberg H. Blennow K.. (2019). Biomarker-guided clustering of Alzheimer's disease clinical syndromes. Neurobiol. Aging 83, 42–53. 10.1016/j.neurobiolaging.2019.08.032 31585366

[B93] Tsatsanis A. Wong B. X. Gunn A. P. Ayton S. Bush A. I. Devos D.. (2020). Amyloidogenic processing of Alzheimer's disease β-amyloid precursor protein induces cellular iron retention. Mol. Psychiatry 25, 1958–1966. 10.1038/s41380-020-0762-0 32444869

[B94] Vermunt L. Sikkes S. A. M. van den Hout A. Handels R. Bos I. van der Flier W. M.. (2019). Duration of preclinical, prodromal, and dementia stages of Alzheimer's disease in relation to age, sex, and APOE genotype. Alzheimers. Dement. 15, 888–898. 10.1016/j.jalz.2019.04.001 31164314 PMC6646097

[B95] Villemagne V. L. Pike K. E. Darby D. Maruff P. Savage G. Ng S.. (2008). Abeta deposits in older non-demented individuals with cognitive decline are indicative of preclinical Alzheimer's disease. Neuropsychologia 46, 1688–1697. 10.1016/j.neuropsychologia.2008.02.008 18343463

[B96] Ward J. H. Jr. (1963). Hierarchical grouping to optimize an objective function. J. Am. Stat. Assoc. 58, 236–244. 10.1080/01621459.1963.10500845

[B97] Ward R. J. Zucca F. A. Duyn J. H. Crichton R. R. Zecca L. (2014). The role of iron in brain ageing and neurodegenerative disorders. Lancet Neurol. 13, 1045–1060. 10.1016/S1474-4422(14)70117-6 25231526 PMC5672917

[B98] Yamamoto A. Shin R.-W. Hasegawa K. Naiki H. Sato H. Yoshimasu F.. (2002). Iron (III) induces aggregation of hyperphosphorylated tau and its reduction to iron (II) reverses the aggregation: implications in the formation of neurofibrillary tangles of Alzheimer's disease. J. Neurochem. 82, 1137–1147. 10.1046/j.1471-4159.2002.t01-1-01061.x 12358761

[B99] Zecca L. Youdim M. B. H. Riederer P. Connor J. R. Crichton R. R. (2004). Iron, brain ageing and neurodegenerative disorders. Nat. Rev. Neurosci. 5, 863–873. 10.1038/nrn1537 15496864

