# Supplementary material for: Machine Learning Profiling of Alzheimer's Disease Patients Based on Current Cerebrospinal Fluid Markers and Iron Content in Biofluids
Source: Front Aging Neurosci. 2021 Feb 22;13:607858. doi: 10.3389/fnagi.2021.607858 (PMC7937894; doi:10.3389/fnagi.2021.607858)
Supplement: Supplementary file 1 [file Table_1.DOCX]

Supplementary Material

# Supplementary Data

**Participants**

Control group (N=14) included patients with suspected polyneuropathy (N=5), muscular dystrophy (N=1), vascular encephalopathy (N=3), multiple sclerosis (N=5).

All patients underwent lumbar puncture with determination of CSF levels of Aβ42, p-Tau and t-Tau.

All CSF samples were obtained early in the morning after overnight fasting by lumbar puncture using an atraumatic needle(Doherty and Forbes, 2014).

CSF samples were collected in polypropylene tubes using standard sterile techniques. CSF samples were centrifuged to eliminate cells and cellular debris, and immediately frozen at -80°C to measure orexin, t-Tau, p-Tau and Aβ42 levels.

CSF Aβ42, t-Tau and p-Tau levels were determined using commercially available sandwich enzyme-linked immunosorbent assays (ELISA; Innotest b-Amyloid 1-42, Innotest h-T-tau, Innotest Phospho-T-tau 181; Fujirebio Ghent, Belgium). All the samples were analysed in duplicate.

Blood samples were collected in polypropylene tubes using standard sterile techniques.

Serum samples were obtained from the removal of the clot by centrifuging whole blood samples.

Transferrin in serum (s-Tf) was evaluated using immunoturbidimetryoptimized with polyethylene glycol (ADVIA Chemistry TRF Transferrin Reagent). Range of normality for s-Tf = [200 – 330] mg/dL.

Doherty, C. M., and Forbes, R. B. (2014). Diagnostic Lumbar Puncture. *Ulster Med J* 83, 93–102.

**Iron determination in CSF using GF-AAS**

The determination of iron in CSF samples was carried out in a controlled atmosphere laboratory provided with filtered air and laminar flow hoods, adopting all precautions to avoid sample contaminations arising from vessels, reagents, and handling. Total iron was dosed in CSF using a Perkin Elmer Analyst 600 Graphite Furnace Atomic Absorption Spectrometer (GF-AAS), equipped with an autosampler, THGA (transverse heated graphite atomizer), and Zeeman-effect background correction. A hollow cathode lamp (Perkin-Elmer) was used for iron, setting the absorption wavelength at 248,3 nm. Pyrocoated graphite platform-integrated tubes were used throughout. The graphite furnace temperature program designed for the atomization of the analyte of interest, consisting of four-steps, is reported (Table S1).

High-purity water (HPW) with a specific resistivity of 18 MΩ cm^−1^, obtained with a Milli-Q water system (Millipore, Bedford, MA), was used for the preparation of the standard and dilution of the samples. All chemicals employed in this work were of analytical grade purity. Iron standard solutions were prepared from a concentrated stock solution of 1000 mg/L iron (Sigma Aldrich). Blank solutions were prepared using HPW with 0.5% nitric acid.

Magnesium Nitrate Mg(NO_3_)_2_ (Sigma Aldrich) was used as matrix modifier (15 µg diluted in 10 mL of HPW) to improve the absorbance signal. The instrument was calibrated daily using freshly prepared iron standard solutions of concentration 5, 10, 15, 20 μg/L. The autosampler tubes were cleaned between measurements by aspirating aliquots of HPW with 0.1% Triton X-100.

After a minimum pre-treatment of CSF samples (1 mL diluted 1:3) iron was evaluated by means of the Standard Addition Method, using two additions for each sample. Concentration and absorbance were used to obtain the calibration for each sample and the final concentration of iron was estimated from the intercept of line calibration, corrected for the dilution factor. Measurements with R^2^ > 0.97 were accepted.

# 2 Supplementary Figures and Tables

| Step | Temperature |  | Ramp time | Hold Time |
| --- | --- | --- | --- | --- |
| Drying | 130° | Removal of acqueous component | 15 s | 30 s |
| Pyrolisis | 1400° | Removal of organic matter and volatil component | 10 s | 20 s |
| Atomization | 2100° | Atomization of analyte | 0 s | 3 s |
| Cleaning | 2450° | Clean up of the furnace | 1 s | 3 s |

**Supplementary Table 1.** Four steps graphite furnace temperature program designed for the atomization of the analyte of interest (iron).

**Graphical representation of variables (Age, Biomarkers, s-Tf , MMSE)**
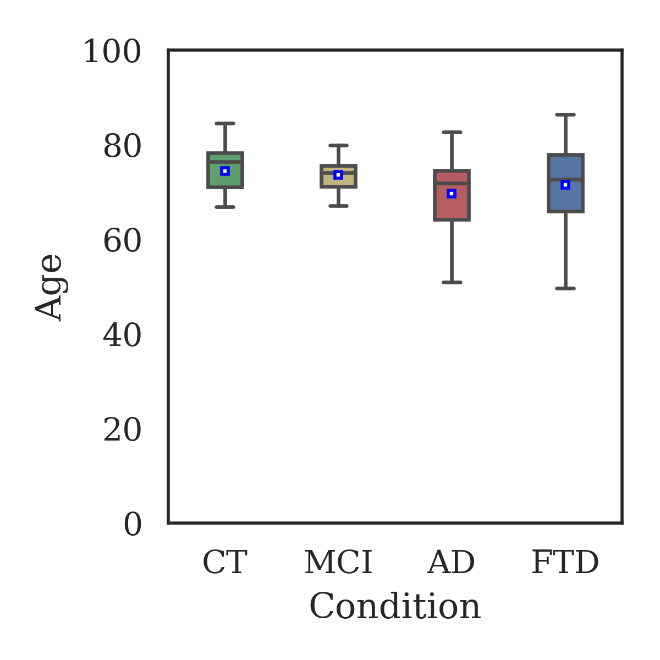

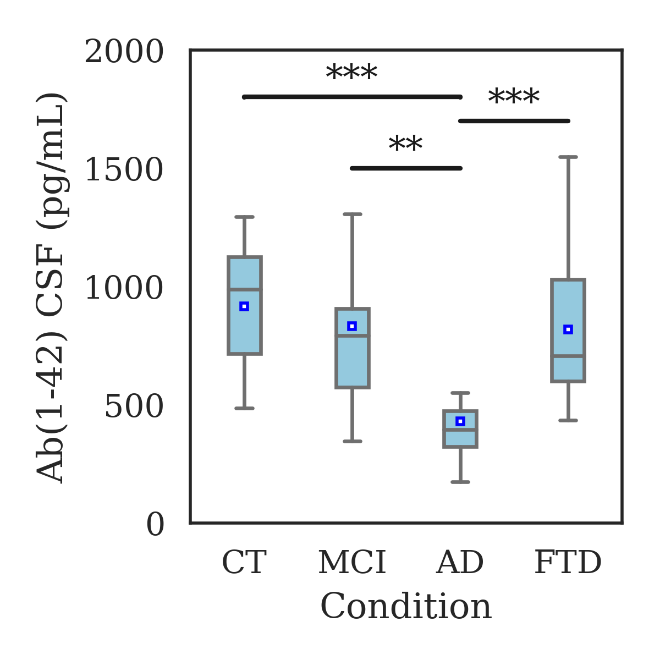


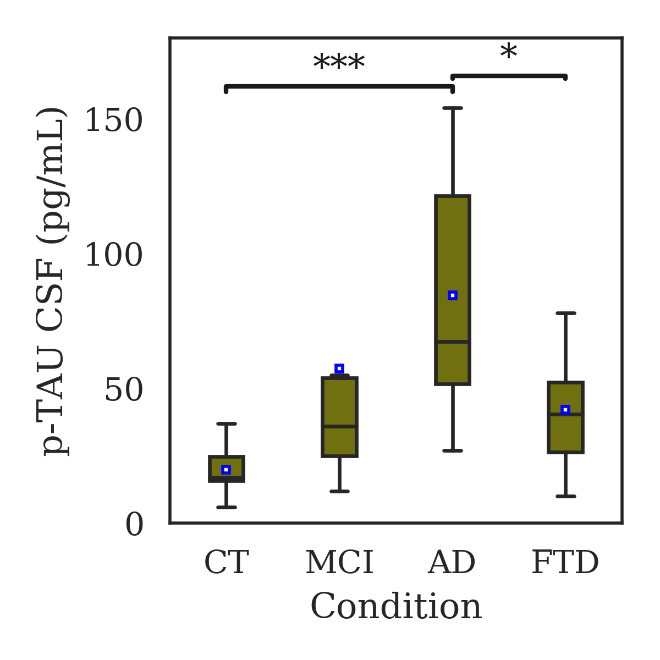

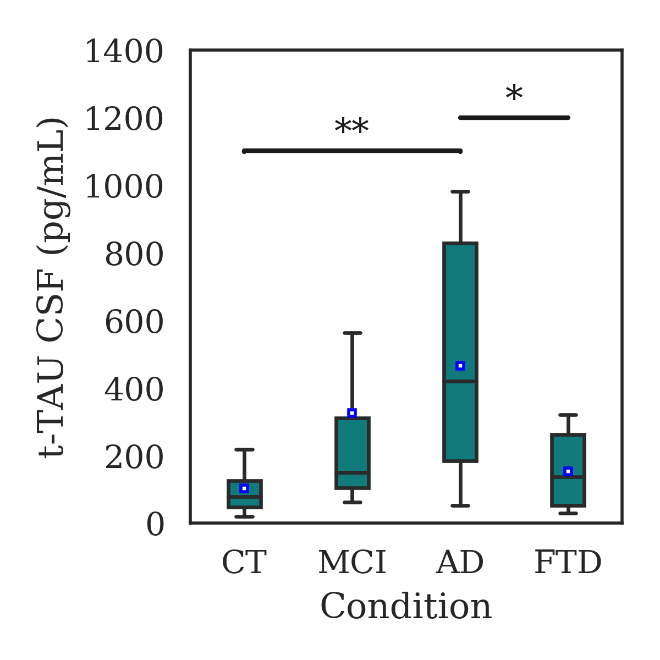


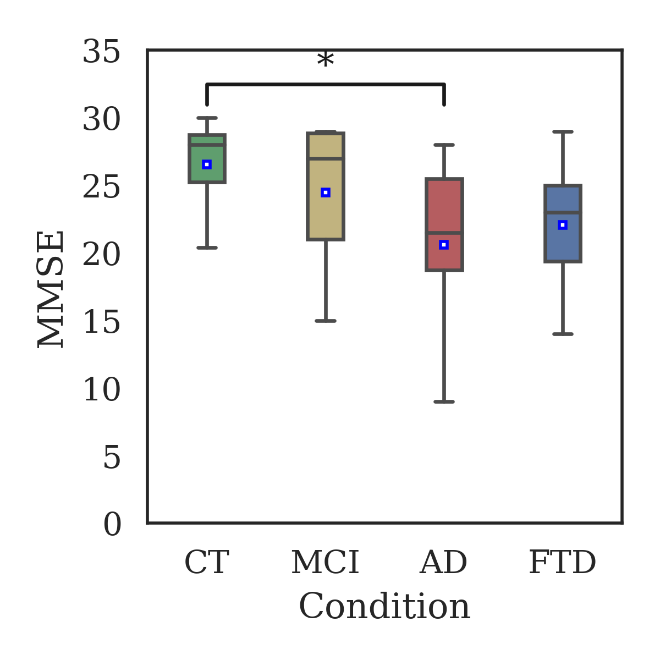

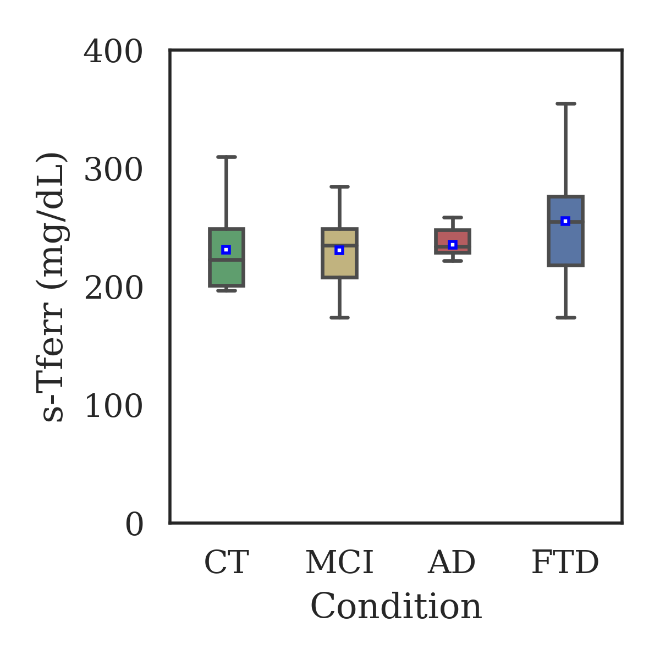


**Supplementary Figure 1.** Boxplot for variables whose value is reported in Table 1. Significant differences have been indicated (* p<0.05; ** p<0.01; *** p< 0.001).

In addition, we reported significant associations satisfying the criteria of Spearman coefficient |r_s_|>0.5, reflecting a non-parametric relationship between the variables. Considering the population of CT, MCI, and AD patients, we found a positive association between iron CSF and p-Tau (r_s_= 0.53, p<0.001), and in the subpopulation in which s-Tf is available a negative association between iron CSF and Aβ42 (r_s_= -0.54, p=0.003). A weak negative association has been found between s-Tf and age (r_s_= -0.45, p= 0.014). In AD group, s-Tf showed a negative correlation with age (r_s_= -0.63, p= 0.037). For the FTD patients, a negative association has been found between p-Tau and Aβ42 (rs= -0.56, p=0.007).

**Clustering Analysis**

After the application of hierarchical clustering for different set of features, values of features within each cluster are reported.

1. **Clustering Standard Biomarkers (SBs) (p-Tau, Aβ42);**

| **Cluster** | **p-Tau**  **Mean ± sd** | **p-Tau**  **Median** | **Aβ42**  **Mean ± sd** | **Aβ42**  **Median** |
| --- | --- | --- | --- | --- |
| 1 | (144.2±29.2) | 147.8 | (469.7±185.6) | 432.5 |
| 2 | (20.1±9.8) | 18.5 | (1229.0±228.5) | 1165.0 |
| 3 | (37.1±16.1) | 36.0 | (578.9±216.67) | 534.0 |

**Supplementary Table 2.** Biomarkers values in each cluster using the features set SBs.

**b) Clustering SBs+ CSF iron**

| **Cluster** | **p-Tau**  **Mean ± sd** | **p-Tau**  **Median** | **Aβ42**  **Mean ± sd** | **Aβ42**  **Median** | **Fe CSF**  **Mean ± sd** | **Fe CSF**  **Median** |
| --- | --- | --- | --- | --- | --- | --- |
| 1 | (33.0±19.1) | 25.0 | (512.7±98.5) | 497.0 | (27.7±9.2) | 28.5 |
| 2 | (27.2±15.5) | 24.0 | (1091.2±264.5) | 1081.0 | (27.7±9.2) | 24.9 |
| 3 | (156.5±23.3) | 154.2 | (489.1±214.2) | 473.0 | (48.6±11.0) | 50.0 |
| 4 | (58.4 ±37.4) | 43.2 | (428.9±146.4) | 403.0 | (67.7±13.8) | 65.2 |

**Supplementary Table 3.** Biomarkers values in each cluster using the features set SBs and iron concentration in CSF.

1. **Clustering SBs in the subpopulation**

Hierarchical clustering in the subpopulation N=29, in which s-Tf is available, using set of features comprising p-Tau and Aβ. Three clusters (sizes: N=7, N=12, N=10) are emerged (Figure S3). Differences in Aβ was very significant (p<0.001) between clusters 1, while differences in p-Tau concentration was highly significant (p<0.001) between clusters 1 and 3 and clusters 1 and 3 (p=0.005). External scores have been evaluated for the clustering: V-measure (0.26), ARI (0.12), AMI (0.20).


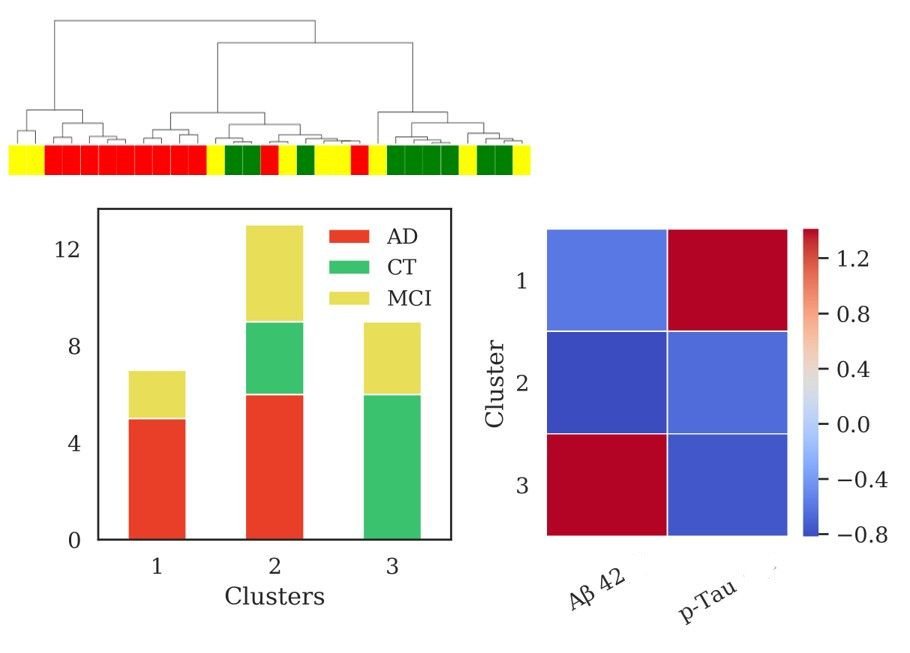


**Supplementary Figure 2.** Results of hierarchical clustering using standard biomarkers Aβ and p-Tau. Left: Dendrogram (yellow =MCI; red =AD; green=CT) and distribution of patients within the three clusters. Right: Heatmap using the median value of the features (Z-score unit) in each cluster. (AD= Alzheimer’s Disease; CT = neurological control; MCI = Mild Cognitive Impairment).

| **Cluster** | **p-Tau**  **Mean ± sd** | **p-Tau**  **Median** | **Aβ42**  **Mean ± sd** | **Aβ42**  **Median** |
| --- | --- | --- | --- | --- |
| 1 | (144.7±29.4) | 154.0 | (477.6±217.4) | 392.0 |
| 2 | (33.6±18.03) | 27.0 | (490.8±170.4) | 486.0 |
| 3 | (22.0±9.87) | 21.0 | (1272.3±246.0) | 1283.0 |

**Supplementary Table 4.** Biomarkers values in each cluster using the features set SBs in the subpopulation in which s-Tf is available.

1. **Clustering SBs+ CSF iron in the subpopulation**

Hierarchical clustering in the subpopulation N=29, in which s-Tf is available, using set of features comprising p-Tau, Aβ and iron concentration in CSF. The addition of iron dosage in CSF unraveled four clusters (sizes: N=5, N=9, N=6, N=9) after the application of hierarchical clustering algorithm, reported in Figure S5. The clusters composed of AD and MCI patients (cluster 3 and cluster 4) significantly differed among them for p-Tau (p<0.001) profile. Cluster 3 differed from cluster 2 (mainly composed of CT patients) for p-tau with high significance (p<0.001), while they differ from cluster 1 for iron content (p=0.04) and p-Tau (p<0.001).

The addition of CSF iron levels improved V-measure (0.40), ARI (0.25) and AMI (0.33) with respect to the same scores obtained with the biomarkers set, described in the previous section.


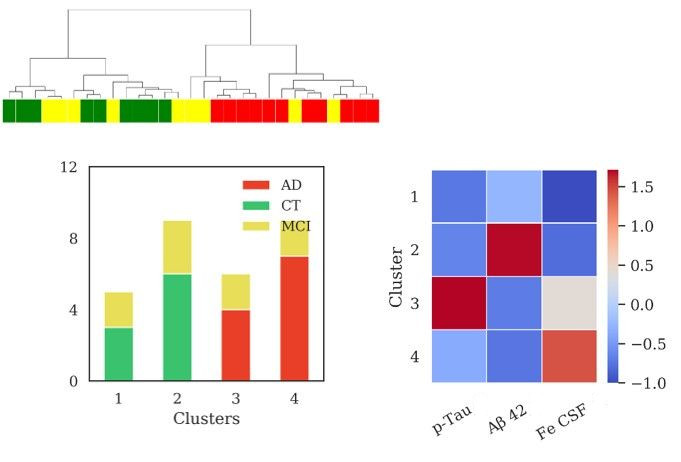


**Supplementary Figure 3.** Results of hierarchical clustering using biomarkers and iron concentration in CSF. Left: Dendrogram (yellow =MCI; red =AD; green=CT) and distribution of patients within the four clusters. Right: Heatmap using the median value of the features (Z-score unit) in each cluster. (AD= Alzheimer’s Disease; CT = neurological control; MCI = Mild Cognitive Impairment).

| **Cluster** | **p-Tau**  **Mean ± sd** | **p-Tau**  **Median** | **Aβ42**  **Mean ± sd** | **Aβ42**  **Median** | **Fe CSF**  **Mean ± sd** | **Fe CSF**  **Median** |
| --- | --- | --- | --- | --- | --- | --- |
| 1 | (19.6±5.0) | 17.0 | (603.4±121.1) | 576.0 | (25.4±8.5) | 26.6 |
| 2 | (22.0±9.9) | 21.0. | (1272.3±246.0) | 1283.0 | (31.4±10.8) | 30.3 |
| 3 | (152.6±22.8) | 154.1 | (491.8±234.5) | 420.0 | (47.7±11.7) | 47.8 |
| 4 | (48.5 ±24.8) | 37.3 | (417.2±153.1) | 406.0 | (59.6±12.4) | 62.6 |

**Supplementary Table 5.** Biomarkers values in each cluster using the features set SBs and iron concentration in CSF for the subpopulation in which s-Tf is available.

**e) Clustering SBs+ s-Tf**

| **Cluster** | **p-Tau**  **Mean ± sd** | **p-Tau**  **Median** | **Aβ42**  **Mean ± sd** | **Aβ42**  **Median** | **s-Tf**  **Mean ± sd** | **s-Tf**  **Median** |
| --- | --- | --- | --- | --- | --- | --- |
| 1 | (144.7±29.4) | 154.0 | (446.6±161.6) | 392.0 | (220.0±16.5) | 226.0 |
| 2 | (23.5±11.7) | 26.0 | (1334.7±275.8) | 1295.5 | (263.0± 25.3) | 256.5 |
| 3 | (39.5±18.8) | 32.0 | (477.6±217.4) | 471.0 | (247.1±18.6) | 240.0 |
| 4 | (19.7±4.5) | 17.0 | (829.14±328.84) | 753.0 | (201.3±14.7) | 201.0 |

**Supplementary Table 6.** Biomarkers values in each cluster using the features set SBs and s-Tf.

**f) Clustering SBs+ s-Tf+ CSF iron**

| **Cluster** | **p-Tau**  **Mean ± sd** | **p-Tau**  **Median** | **Aβ42**  **Mean ± sd** | **Aβ42**  **Median** |
| --- | --- | --- | --- | --- |
| 1 | (45.1±17.3) | 37.3 | (422.4±176.3) | 449.0 |
| 2 | (144.7±29.4) | 154.0 | (477.6±217.4) | 392.0 |
| 3 | (19.1±4.4) | 16.5 | (786.3±327.7) | 729.0 |
| 4 | (23.7±10.7) | 25.0 | (1226.3±381.6) | 1283.0 |
| **Cluster** | **Fe CSF**  **Mean ± sd** | **Fe CSF**  **Median** | **s-Tf**  **Mean ± sd** | **s-Tf**  **Median** |
| 1 | (57.49±9.19) | 62.58 | (244.14±12.73) | 240.0 |
| 2 | (52.9±17.46) | 49.97 | (220.0±16.49) | 226.0 |
| 3 | (31.24±12.84) | 29.38 | (204.88±16.97) | 203.0 |
| 4 | (29.94±9.88) | 30.02 | (266.14±24.53) | 264.0 |

**Supplementary Table 7.** Biomarkers values in each cluster using full features set: SBs, CSF iron and s-Tf.

**Additional clustering analysis (s-Tf corrected for age by means of linear regression)**

In our population only s-Tf showed some significant association with age (r_s_= -0.45, p=0.014, not significant Pearson’s correlation) in the subpopulation including data of s-Tf (N=29 patients), and within group in CT (r=-0.68, p=0.046, not significant Spearman’s test) and AD (r_s_= -0.63, p=0.04, not significant Pearson’s correlation). To be more accurate, we applied the correction for age for this variable using a linear regression model (p=0.03, R^2^=0.15). The application of HAC using the features set comprising biomarkers and s-Tf (corrected for age) revealed four clusters (sizes: N=6, N=5, N=9, N=9), reported in Supplementary Figure 4. Significant differences among clusters were found for the p-Tau values when comparing clusters 1and 3 (p=0.004) and 1 and 4 (p<0.001); s-Tf differed between clusters 2 and 3 (p=0.003); Aβ42 was significantly different when comparing clusters 1 and 4, 2 and 4,3 and 4 (p<0.001). Clustering scores showed the following values: V-measure (0.36), ARI (0.18), AMI (0.28).


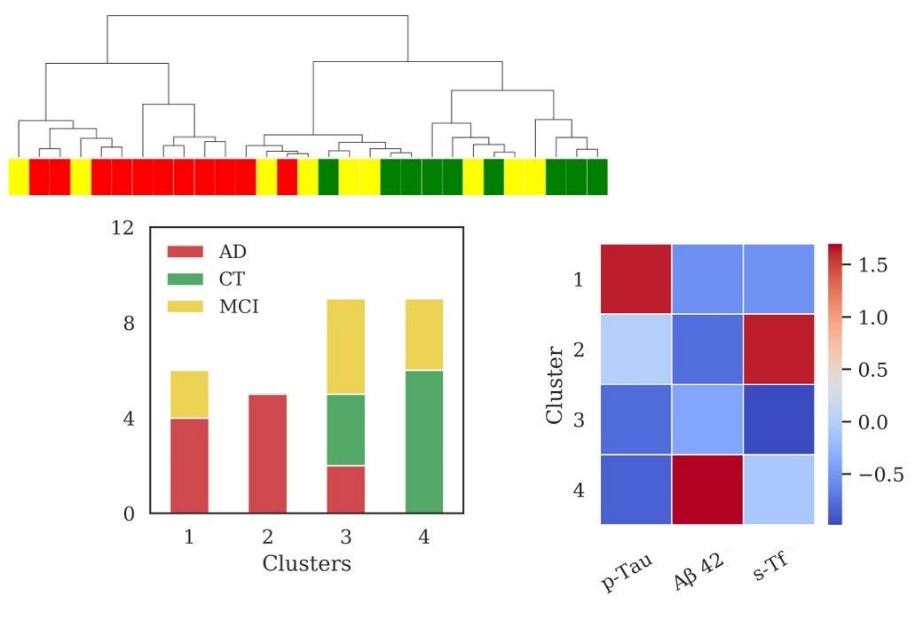


**Supplementary Figure 4.** Results of hierarchical clustering using biomarkers and s-Tf (corrected for age). Left: Dendrogram (yellow =MCI; red =AD; green=CT) and distribution of patients within the four clusters. Right: Heatmap using the median value of the features (Z-score unit) in each cluster. Abbreviations: AD= Alzheimer’s Disease; CT = neurological control; MCI = Mild Cognitive Impairment

Considering all the features (biomarkers, s-Tf, and iron CSF) we reported two different situations:

1. Number of resulting clusters=5 (set finding clustering step where the acceleration of distance growth is the largest). The five clusters (size: N=6, N=8, N=5, N=5, N=5), reported in Supplementary Figure 5. Significant differences among clusters were found for the p-Tau values when comparing clusters 1and 3 (p=0.01),2 and 3 (p<0.001) and 2 and 5 (p=0.04); s-Tf differed between clusters 1 and 2, and 3 and 5 (p=0.01), 2 and 5 (p<0.001) and 4 and 5 (p=0.003); Aβ42 was significantly different when comparing clusters 1 and 3(p=0.04), 1 and 4 (p=0.02),1 and 5 (p=0.002); iron CSF differed between cluster 1 and 4 (p=0.013), 2 and 4 (p=0.002), 2 and 5 (p=0.017). Clustering scores showed the following values: V-measure (0.42), ARI (0.22), AMI (0.34). The (Fe CSF/ s-Tf) ratio in cluster 1 (0.14) and cluster 2 (0.12) is lower with respect to cluster 3 (0.22), cluster 4 (0.27) and cluster 5 (0.20), in which the ratio is increased.


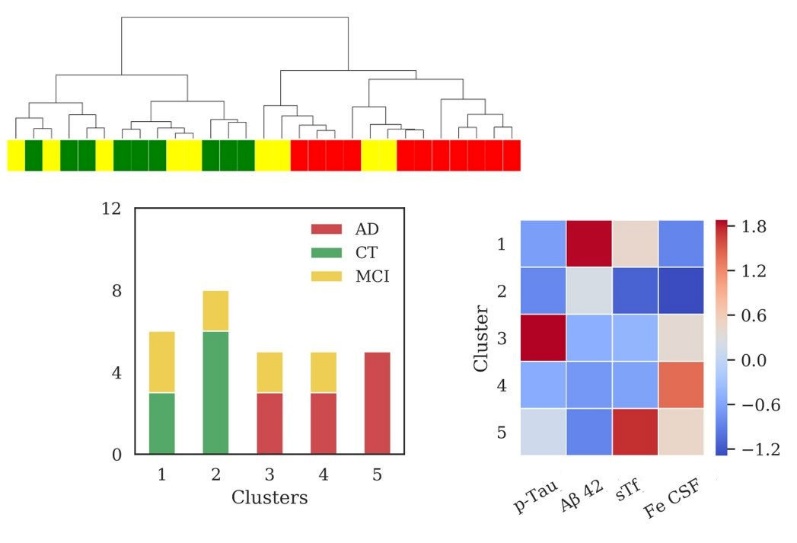


**Supplementary Figure 5.** Results of hierarchical clustering using biomarkers, iron CSF and s-Tf (corrected for age). Left: Dendrogram (yellow =MCI; red =AD; green=CT) and distribution of patients within the four clusters. Right: Heatmap using the median value of the features (Z-score unit) in each cluster. Abbreviations: AD= Alzheimer’s Disease; CT = neurological control; MCI = Mild Cognitive Impairment

1. Number of clusters =4, set manually in order to better compare with our previous analysis. The four clusters (size: N=6, N=8, N=5, N=10), reported in Supplementary Figure 5. Cluster 3 and cluster 4 are composed only of AD and MCI patients. One of these clusters (cluster 4) presented a significant difference in the levels of s-Tf (p=0.004), Iron CSF (p<0.001), p-Tau (p=0.03), and Aβ42 (p=0.013) with respect to cluster 2 (mainly CT patients). Cluster 3 differed from cluster 1 (composed only by MCI and CT patients) in the biomarkers (for Aβ42 p=0.004; for p-Tau p=0.007) ad from cluster 2 for p-Tau (p<0.001). Clusters 1 and 2 significantly differed for Aβ42 (p=0.04) and for s-Tf (p=0.008). Finally, cluster 1 differed from cluster 4 for the Aβ42 and iron CSF values (p <0.001). Clustering scores showed the following values: V-measure (0.42), ARI (0.30), AMI (0.35). The (Fe CSF/ s-Tf) ratio in cluster 1 (0.14) and cluster 2 (0.12) is lower with respect to cluster 3 (0.22) and cluster 4 (0.25) in which the ratio is increased.


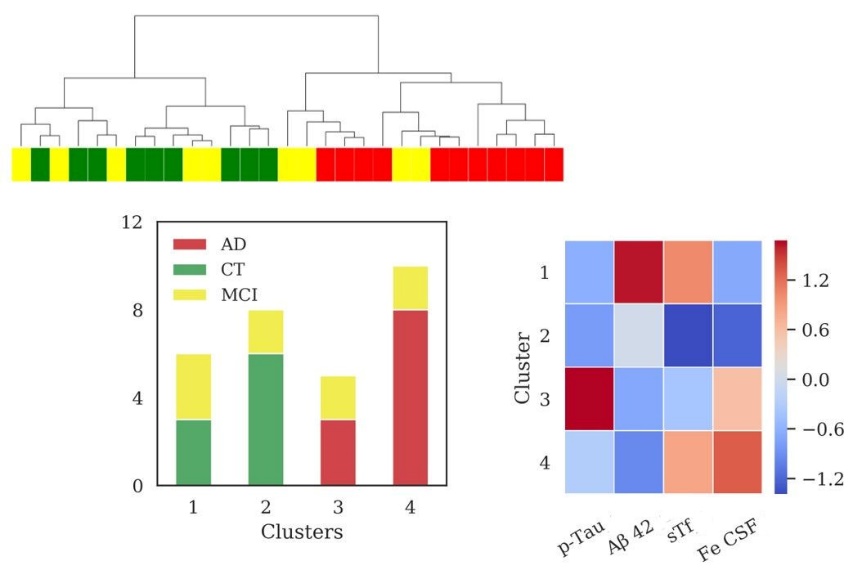


**Supplementary Figure 6.** Results of hierarchical clustering using biomarkers, iron CSF and s-Tf (corrected for age), setting number of clusters=4. Left: Dendrogram (yellow =MCI; red =AD; green=CT) and distribution of patients within the four clusters. Right: Heatmap using the median value of the features (Z-score unit) in each cluster. Abbreviations: AD= Alzheimer’s Disease; CT = neurological control; MCI = Mild Cognitive Impairment

**Description of the population of patients**

|  | **Mean** | **Median** | **Lower Quartile** | **Upper quartile** |
| --- | --- | --- | --- | --- |
| **Aβ42CSF (pg/mL)** | 721.7 | 582.0 | 455.0 | 989.0 |
| **p-Tau CSF (pg/mL)** | 55.5 | 36.0 | 22.0 | 60.5 |
| **t-Tau CSF (pg/mL)** | 307.2 | 151.0 | 78.5 | 421.0 |
| **Fe CSF (μg/L)** | 41.0 | 37.2 | 23.6 | 53.1 |

**Supplementary Table 8.** Description of the population of patients (N=47)

|  | **Mean** | **Median** | **Lower Quartile** | **Upper quartile** |
| --- | --- | --- | --- | --- |
| **Aβ42 CSF (pg/mL)** | 730.1 | 576.0 | 406.0 | 1059.0 |
| **p-Tau CSF (pg/mL)** | 56.8 | 31.0 | 21.0 | 69.8 |
| **t-Tau CSF (pg/mL)** | 322.8 | 151.0 | 77.0 | 426.0 |
| **Fe CSF (μg/L)** | 42.5 | 41.0 | 29.8 | 52.1 |
| **s-Tf (mg/dL)** | 232.8 | 231.0 | 208.0 | 249.0 |

**Supplementary Table 9.** Description of the subpopulation (in which s-Tf is available) of patients (N=29).

| Cluster | Abeta | p-tau | Fe CSF | s-Tf |
| --- | --- | --- | --- | --- |
| 1 | ↓ | ↑ | ↑↑ | ↑ |
| 2 | ↓↓ | ↑↑ | ↑ | ↓ |
| 3 | ↑ | ↓↓ | ↓↓ | ↓↓ |
| 4 | ↑↑ | ↓ | ↓ | ↑↑ |

**Supplementary Table 10.** Cluster profiles for the full set features (biomarkers, Fe CSF, s-Tf) according to the quartile (computed on the subpopulation) in which the median value of the variable of each cluster falls. ↓↓: under lower quartile; ↓: under median; ↑: up median; ↑↑: up upper quartile.
